# Delta opioid peptide [D-ala2, D-leu5]-Enkephalin’s ability to enhance mitophagy via TRPV4 to relieve ischemia/reperfusion injury in brain microvascular endothelial cells

**DOI:** 10.1136/svn-2023-003080

**Published:** 2024-05-02

**Authors:** Zhongfang Deng, Xiaoyu Chen, Ran Zhang, Lingchao Kong, Yang Fang, Jizheng Guo, Bing Shen, Lesha Zhang

**Affiliations:** 1 Department of Physiology, Anhui Medical University, Hefei, Anhui, China; 2 Department of Orthopedics, The First Affiliated Hospital of Anhui Medical University, Hefei, Anhui, China; 3 Department of Pathophysiology, Anhui Medical University, Hefei, Anhui, China; 4 Dr. Neher's Biophysics Laboratory for Innovative Drug Discovery, State Key Laboratory of Quality Research in Chinese Medicine, Macau University of Science and Technology, Taipa, Macao

**Keywords:** ischemic attack, transient, brain, cerebrovascular disorders, pharmacology

## Abstract

**Background:**

Local brain tissue can suffer from ischaemia/reperfusion (I/R) injury, which lead to vascular endothelial damage. The peptide δ opioid receptor (δOR) agonist [D-ala2, D-leu5]-Enkephalin (DADLE) can reduce apoptosis caused by acute I/R injury in brain microvascular endothelial cells (BMECs).

**Objective:**

This study aims to explore the mechanism by which DADLE enhances the level of mitophagy in BMECs by upregulating the expression of transient receptor potential vanilloid subtype 4 (TRPV4).

**Methods:**

BMECs were extracted and made to undergo oxygen-glucose deprivation/reoxygenation (OGD/R) accompanied by DADLE. RNA-seq analysis revealed that DADLE induced increased TRPV4 expression. The CCK-8 method was used to assess the cellular viability; quantitative PCR (qPCR) was used to determine the mRNA expression of *Drp1*; western blot was used to determine the expression of TRPV4 and autophagy-related proteins; and calcium imaging was used to detect the calcium influx. Autophagosomes in in the cells’ mitochondria were observed by using transmission electron microscopy. ELISA was used to measure ATP content, and a JC-1 fluorescent probe was used to detect mitochondrial membrane potential.

**Results:**

When compared with the OGD/R group, OGD/R+DADLE group showed significantly enhanced cellular viability; increased expression of TRPV4, Beclin-1, LC3-II/I, PINK1 and Parkin; decreased p62 expression; a marked rise in calcium influx; further increases in mitophagy, an increase in ATP synthesis and an elevation of mitochondrial membrane potential. These protective effects of DADLE can be blocked by a TRPV4 inhibitor HC067047 or RNAi of TRPV4.

**Conclusion:**

DADLE can promote mitophagy in BMECs through TRPV4, improving mitochondrial function and relieving I/R injury.

**WHAT IS ALREADY KNOWN ON THIS TOPIC**

- δ opioid receptor (δOR) agonist [D-ala2, D-leu5]-Enkephalin (DADLE) can relieve acute ischaemia/reperfusion (I/R) injury in brain microvascular endothelial cells (BMECs). However, its specific mechanisms remain unexplored.

**WHAT THIS STUDY ADDS**

- In this study, the signalling of the increased DADLE-triggered expression of transient receptor potential vanilloid subtype 4 (TRPV4) for improving mitochondrial function in BMECs under oxygen-glucose deprivation/reoxygenation conditions was investigated. DADLE upregulates autophagy and mitophagy levels, raises calcium influx mediated by TRPV4, increases ATP synthesis, elevates mitochondrial membrane potential and then enhances cellular viability. These protective effects of DADLE can be blocked by halting the function or expression of TRPV4.

**HOW THIS STUDY MIGHT AFFECT RESEARCH, PRACTICE OR POLICY**

- This study explains the molecular mechanisms of δOR agonists in improving endothelial function against I/R injury. It provides support for the exploration of new drug targets and the clinical application of δOR agonists as cellular protectants.

## Introduction

 Stroke is the second leading cause of death globally, the majority of which are ischaemic strokes.^1^ Once stroke occurs, the brain suffers from ischaemic hypoxia injury and thrombolysis leads to reperfusion injury. The peptide agonist of the δ opioid receptor (δOR), [D-ala2, D-leu5]-Enkephalin (DADLE), can exert a tissue-protective effect.^2^ Studies have shown that after the administration of DADLE to the brain, δORs are activated, promoting neuron survival.^3^ Our previous studies first discovered an endogenous δOR expression on brain microvascular endothelial cells (BMECs), showing that DADLE can reduce BMECs’ apoptosis caused by ischaemia/reperfusion (I/R).^4^ However, this underlying mechanism has not yet been thoroughly studied.

Mitochondria participate in many intracellular signalling pathways. They have become therapeutic targets for relieving I/R injury of several organs, such as the brain, heart and kidney.^5 6^ Mitophagy is a type of selective autophagy specifically directed toward damaged mitochondria and considered to play a crucial role in the pathophysiological processes of I/R.^7^ Recent studies have shown that the regulation of mitophagy is related to the neuroprotective effect in brain I/R.^6^

Transient receptor potential (TRP) superfamily channels are membrane proteins that serve as non-selective cation channels controlling calcium (Ca^2+^) permeation into cells.^8^ Among those channels, TRP subfamily V member 4 (TRPV4) has been reported to have widespread expression in the human brain^9^ and the vascular endothelial cells of rats.^10^ Studies have shown that activating TRPV4 can help patients who underwent ischaemic stroke to recover by promoting vascular regeneration.^11^ Calcium ions, the second messenger, are involved in regulating cellular apoptosis and autophagy.^12^ The Ca^2+^ inflow mediated by TRPV4 can respond to various stimuli to maintain intracellular Ca^2+^ stability.^13 14^ The change in the expression level of TRPV4 may directly affect the inflow of Ca^2+^, or interfere the Ca^2+^ entry mediated by store-operated Ca^2+^ entry (SOCE).^15^ However, current research on TRPV4 in BMECs is scarce, and there is even less research on the functional connection among TRPV4, G-protein-coupled and mitophagy. Through RNA-seq analysis, we found that DADLE could induce the upregulation of TRPV4 in BMECs when subjected to oxygen-glucose deprivation/reoxygenation (OGD/R) injury in vitro. Therefore, we hypothesise that DADLE may upregulate the TRPV4-mediated Ca^2+^ inflow to enhance cellular mitophagy level, improve mitochondrial function, and alleviate I/R injuries suffered by BMECs. This study could enrich the molecular mechanisms of δOR agonists improving BMEC against I/R injury, and lend support to the clinical application of δOR agonists as cellular protectants.

## Methods

### Cell culture of BMECs and treatment

Five extractions were performed. For every extraction, four Sprague Dawley rats (provided by the Anhui Medical University Animal Centre, 3 weeks of age, weighing 20 g, two males and two females) were euthanised by making them over-inhale CO_2_. BMECs were isolated and cultured according to the previously described method.^4^ The cells were cultured at 37℃ with 5% CO_2_ in a endothelial cell culture medium from Pricells (Hubei, China) containing 10% FBS, 1% penicillin/streptomycin, being supplemented with VEGF, EGF, FGF and IGF-1. The δOR agonist, DADLE (E7131, Sigma-Aldrich), the TRPV4 agonist, GSK1016790A (HY-19608, MCE) and the TRPV4 antagonist, HC-067047 (HY-100208, MCE) were dissolved in DMSO.

### Immunofluorescence

The cells were seeded on the 0.01% poly-lysine-coated cover slips overnight. After being fixed with 4% paraformaldehyde, the cells were permeabilised with 0.5% TritonX-100/phosphate-buffered saline (PBS) for 15 min. The primary antibodies were as follows: vWF (1:100, sc-365712, Santa Cruz), δOR (1:200, ab176324, Abcam), aSMA (1:200, Affinity, AF1032), CD31 (1:200, NB100-2284, Novus), GFAP (1:200, sc-33673, Santa Cruz), Iba-1 (1:200, 019–19741, Wako) and VEGF-A (1:200, bs-1313R, Bioss) at 4℃ overnight. The fluorescent secondary antibodies were Alexa Fluor 555 (1:300, A31572, Thermo) and Alexa Fluor 488 (1:300, A21202, Thermo) at 37℃ for 1 hour. The images were captured under either a fluorescence microscope or a confocal microscope.

### Oxygen-glucose deprivation/reoxygenation

Cells were plated in a 12-well plate, and the supernatant was replaced with glucose-free Earle's balanced salt solution (EBSS) (Procell, China). The plate was subsequently placed in a hypoxia incubator (H35 HPEA, UK) with a gas concentration of 0.1% oxygen, 5% carbon dioxide and 94.9% nitrogen, and cultivated for 12 hours at 37℃. Then, the medium was replaced with complete culture medium, and reoxygenated for 6 hours. During the OGD/R process, cells in different groups were respectively exposed to solvent, 5 nM DADLE or 5 nM DADLE plus 5 µM HC067047.

### RNA sequencing (RNA-seq) and transcriptome analysis

RNA libraries were created and sequenced using the Illumina HiSeq 2500 sequencing platform’s high-throughput method to obtain a sequencing length of 150 base pairs. Three RNA sample libraries from the control group, two from the OGD/R group and two from the OGD/R with DADLE group were obtained. All samples had enough quantity required for high-throughput sequencing. The RNA-Seq Fastq raw data were then processed with Trimmomatic to remove adapters and reduce quality reads, then evaluated using the FastQC software. Quality-approved data were aligned with the rat reference genome (National Center for Biotechnology Information (NCBI) genome assembly, version Rnor_6.0) using HISAT2 (V.2.0.13) and annotated with the NCBI Rnor_6.0 .gtf annotation file. Known messenger RNA (mRNA) counts were obtained with the Kallisto RNA-Seq quantification software to compare transcript expression differences. Differences in read numbers between samples were analysed using the edgeR package software (http://bioconductor.org/packages/2.4/bioc/html/edgeR.html). Transcripts showing |fold change|>2 with a p<0.05 were significantly different.

### Western blot

Cells were lysed with RIPA buffer, then placed in a metal bath at 105℃ for 10 min. Equal amounts of protein were electrophoresed on SDS-PAGE gel and wet-transferred onto PVDF membrane. After blocking with 5% non-fat milk in PBST for 1.5 hours, the following primary antibodies were used: TRPV4 (1:1000, DF8624, Affinity); p62 (1:2000, #5114, CST) and LC3A/B (1:2000, #12741, CST); δOR (1:1000, ab176324, Abcam); Beclin1 (1:1000, 11 306-1-AP), PINK1(1:1000, 23 274-1-AP, Proteintech) and Parkin (1:1000, 14 060-1-AP, Proteintech). Wash with PBST buffer three times and then incubate with the HRP-conjugated goat antibody IgG (1:5000, E-AB-1003, Elabscience) at R.T. for 1.5 hours. A chemiluminescent reagent and a gel imaging analysis system were used to analyse the intensity. All data about strips can be found in the online supplemental material.

### Cell viability assay

The viability of the cells was determined using the Enhanced Cell Counting Kit-8 (CCK-8) (C0037, Beyotime). Cells were seeded into a 96-well plate; after 24 hours, they were made to undergo OGD/R with or without drugs. Each well was then incubated with 90 µL of serum-free medium and 10 µL of CCK-8 reagent for 1.5 hour. Finally, the absorbance at 450 nm was recorded.

### Mitochondrial membrane potential measurement

The mitochondrial membrane potential was measured following the instructions of a JC-1 detection kit (M8650-100T, Solarbio). JC-1 is a novel fluorescent probe that, once it enters the mitochondria, accumulates in the matrix to form aggregates at higher mitochondrial membrane potential areas, thus emitting red fluorescence. It keeps monomer form at lower mitochondrial membrane potential areas, emitting green fluorescence. The ratio of red to green fluorescence intensity represents the mitochondrial membrane potential.

### Measurement of ATP content

The ATP content was measured following the instructions of an Enhanced ATP Assay Kit (S0027, Beyotime, China). After reaction, the fluorescence values were measured using a Flexstation (Molecular Devices, China).

### Quantitative real-time PCR

Total mRNA extraction, cDNA conversion and fluorescent quantitative PCR amplification were all performed using the kits (AC0103, AG0302, and AH0101-B, Sparkjade, China). The primers used in qPCR are shown as followed (table 1):

**Table 1:** The primers used in qPCR

Drp1	Forward: 5′-ACAACAGGAGAAGAAAATGGAGT-3′
	Reverse: 5′-ATCCACAAGCGTCAGGTTGA-3′
Actb	Forward: 5′-CATTGTTACCAACTGGGACGACAT-3′
	Reverse: 5′-GCCTCGGTGAGCAGCACA-3′

Relative expression levels were calculated using the 2−△△Ct method, with Actb used to normalizenormalise the relative messengerm RNA expression level of Drp1.

Drp1dynamin-related protein 1qPCRquantitative PCR

### Transmission electron microscopy (TEM)

The cells were fixed with 4% paraformaldehyde and then underwent four washes with PBS, with each wash lasting for 1 hour. After fixation for 2 hours at 4°C, the cells were rinsed three times with distilled water and then underwent gradient dehydration. The cells were then embedded in EPON 812 resin and sectioned at a thickness of 70 nm–100 nm. Finally, the ultrastructure of the cells was observed under a TEM (Talos L120C G2, Thermo, USA).

### Ca^2+^ imaging

After various drug treatments, the cells were incubated with 10 µM Fluo-8/AM in a 37℃ incubator for 30 min. They were then washed twice with PBS and clamped in a chamber with a volume of 500 µL. The N-phenylthiourea sulfonate solution (NPSS) (ddH_2_O: 500 mL, NaCl: 4.099 g, KCl: 0.1862 g, MgCl_2_: 0.0475 g, CaCl_2_: 0.0555 g, Glucose·H_2_O: 0.991 g, HEPES: 0.5957 g, pH=7.4) was added to the chamber. When the calcium ion influx in the cells reached equilibrium, the cells were stimulated with 50 nM GSK1016790A. To detect the SOCE signal, the octylphenol ethoxylate sulfate solution (OPSS), which does not contain CaCl_2_ was used. Thapsigargin (TG) (2 µM) was added to exhaust the calcium stores and activate SOCE. Subsequently, 2 mM CaCl_2_ was added. The changes in calcium signal fluorescence intensity were represented by the ratio of the fluorescence intensity after and before the addition (F1/F0).^16^

### siRNA transfection

The siRNAs were synthesised by Sangon Biotech (Shanghai, China). Their sequences are shown as followed (table 2):

**Table 2:** The sequences of siRNA targeting TRPV4 and scrambled siRNA

siRNA targeting TRPV4	5′-GGAGCTGAACAAGAACTCA-3′^40^
scrambled siRNA	5′-TTCTCCGAACGTGTCACGTdTdT-3′

The brain microvascular endothelial cellsBMECs were transfected with siRNAs using Lipo3000 (Invitrogen).

### Statistical analysis

These data were statistically presented as mean±SEM and graphed using GraphPad Prism V.8.0.2.263 (San Diego, USA). A t-test was performed when comparing only two sets of data. For comparisons involving more than two groups, a one-way analysis of variance followed by Tukey’s multiple comparison test was employed. A two-tailed p^*^<0.05 was considered statistically significant.

## Results

### RNA-seq results indicated that DADLE could elevate the decreased expression of TRPV4 caused by OGD/R in BMECs

**Figure 1:**
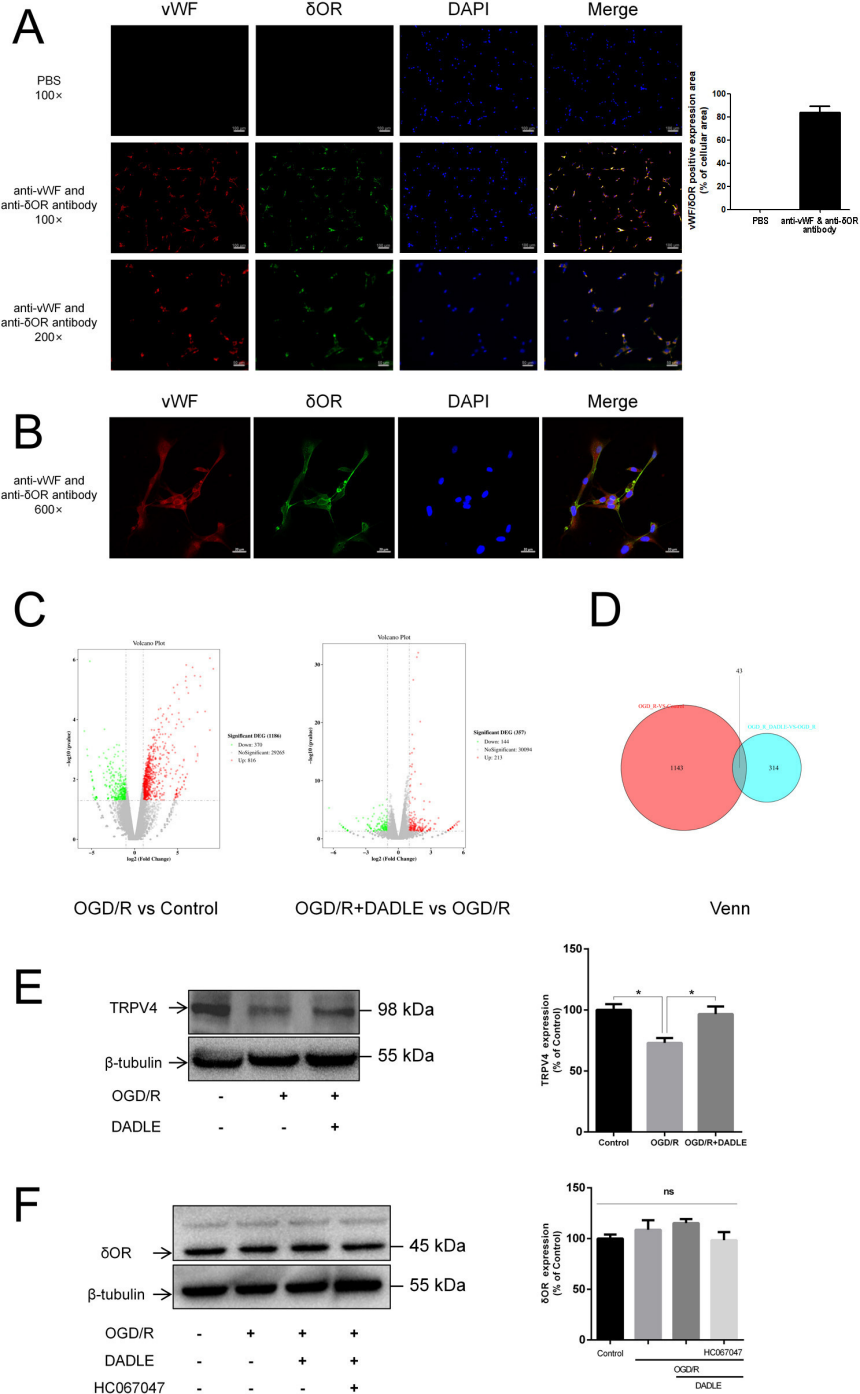
DADLE significantly ameliorates the reduction in TRPV4 expression in primary cultured BMECs caused by OGD/R. (**A**) Expressive detection of δORs in BMECs. Inverted microscope magnification: 100×, scale bar=100 µm; 200×, scale bar=50 µm. Statistical bar shows the ratio of the area of positive dual-staining signal to the area of cells (n=4). (**B**) Localisation of δORs. Laser confocal oil microscope magnification: 600×, scale bar=20 µm (n=3). (**C**) Volcano plot of next-generation high-throughput sequencing (RNAseq): red points represent upregulated genes, and green points represent downregulated genes. (**D**) The Venn diagram shows 43 intersecting genes of the two volcano plots in panel C, including TRPV4. (**E**) Western blotting validated the TRPV4 expressive change (n=4). (**F**) Western blotting examined the effect of different treatments on the expression level of δOR (n=5). Data are presented as mean±SEM and ns indicates no statistical significance, *p<0.05. δOR, δ opioid receptor; BMECs, brain microvascular endothelial cells; DADLE, [D-ala2, D-leu5]-Enkephalin; OGD/R, oxygen-glucose deprivation/reoxygenation; PBS, phosphate-buffered saline; TRPV4, transient receptor potential subfamily V member 4; vWF, von willebrand factor; DAPI, 4',6-diamidino-2-phenylindole.

At the beginning, we harvested the BMECs from three groups: control, OGD/R and OGD/R+DADLE groups. We then analysed the expressive differences among these groups by using next-generation high-throughput RNA-seq. Before sequencing, we carried out morphological observations to identify the expression of vWF and δOR, showing the characteristics of vascular endothelial cells and endogenous δOR expression (figure 1A,B). The ratio of the area of positive dual-staining signal to the area of cells reached 83.82%±4.73%, indicating almost all cells coexpressed vWF and δOR (figure 1A). Figure 1B presents the cellular subregion localisation of vWF and δOR, which were observed by a laser confocal microscope. vWF is mainly distributed in the cytoplasm and δOR on the cell membrane. Another result of immunofluorescence staining of vascular endothelial cell’s markers CD31 and VEGF-A, astrocyte’s marker GFAP, microglia’s marker Iba-1 and pericyte’s marker α-SMA on the unstimulated BMECs of both the first and fifth passage, showed that there is no GFAP-positive, Iba-1-positive and α-SMA-positive signal, but almost all cells present a CD31-positive signal. As VEGF-A, due to its release when cells being stimulated, its expression was less than that of CD-31 (online supplemental figure S1). These results indicated that the BMECs’ purity is acceptable. As the analysis results of RNA-seq show in the volcano diagram (figure 1C), there were 1186 mRNAs worth of existing differences between the OGD/R group and the control group, with 816 upregulated and 370 downregulated genes; while there were 357 mRNAs expressed differently between the OGD/R+DADLE group and the OGD/R group, with 213 upregulated and 114 downregulated genes. The Venn graph showed that intersected 43 kinds of mRNA were shared by the two comparisons, among which TRPV4 showed a decrease in the OGD/R group that could be elevated by DADLE administration (figure 1D). Western blot detection verified this tendency (figure 1E). To exclude the possibility of the downregulated quantity of δOR after stimulation, we detected whether OGD/R, activation of δOR or inhibition of TRPV4 could influence the expression of δOR. In figure 1F, there were no changes in δOR among different groups.

### DADLE could enhance the OGD/R-damaged cellular viability, elevate the mitochondrial membrane potential and promote ATP synthesis

**Figure 2:**
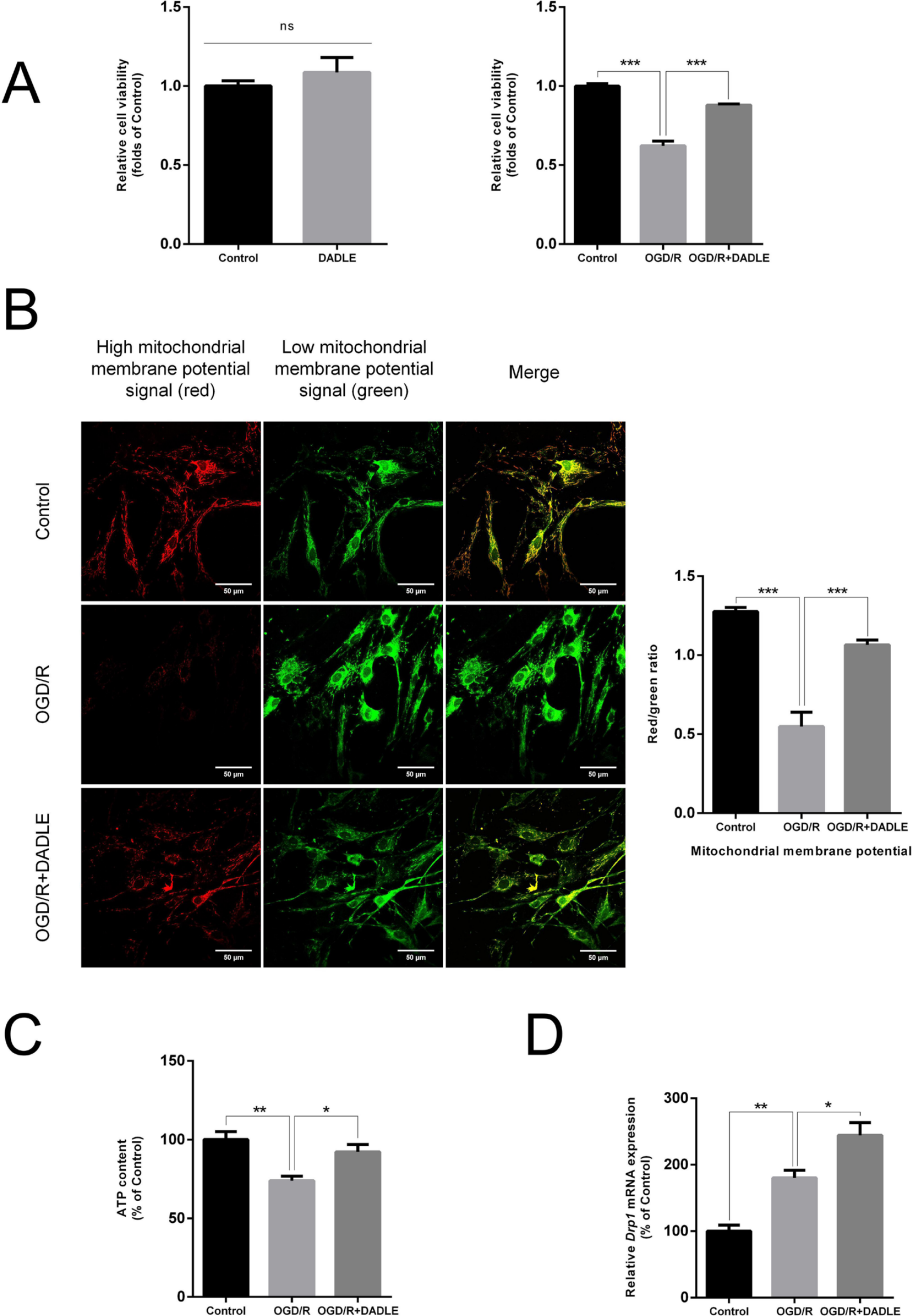
DADLE (5 nM) promotes cell survival damaged by OGD/R injury and improves mitochondrial function. (**A**) CCK8 assay evaluated the relative cell viability of BMECs with concomitant DADLE treatment under normal culture (n=3) and the relative cell viability compared with the OGD/R group after 12 hours of OGD and 6 hours of reperfusion (n=6). (**B**) Changes in the mitochondrial membrane potential levels of BMECs were detected by JC-1 under confocal laser microscopy. Red fluorescence represents JC-1 aggregates accumulating in the mitochondrial matrix with high mitochondrial membrane potential; green fluorescence represents JC-1 monomers diffusing in the cytoplasm of BMECs with low mitochondrial membrane potential (n=9). (**C**) The administration of DADLE improved mitochondrial ATP synthesis (n=7). (**D**) mRNA expression of Drp1 in different groups (n=6). Data are presented as mean±SEM and ns indicates no statistical significance, *p<0.05, **p<0.01, ***p<0.001. BMECs, brain microvascular endothelial cells; DADLE, [D-ala2, D-leu5]-Enkephalin; Drp1, dynamin-related protein 1; mRNA, messenger RNA; OGD/R, oxygen-glucose deprivation/reoxygenation.

It has been reported that TRPV4 is expressed in endothelial cells and modulates mitochondrial activity.^17^ DADLE offers neuroprotection against acute mitochondrial dysfunction.^18^ Therefore, we hypothesise that the protective effect of DADLE on BMECs might be related to mitochondria. Through CCK8 assays, we demonstrated that DADLE does not affect the viability of BMECs under normal conditions, but improves it under OGD/R conditions (figure 2A). Next, we tested changes in the level of mitochondrial membrane potential (figure 2B) and ATP synthesis (figure 2C). Mitochondrial membrane potential, which drives the generation of ATP by mitochondria, changes can be used for assessing the mitochondrial function.^19^ Compared with the control group, the OGD/R group exhibited weak red fluorescence intensity. However, the cells treated with DADLE under OGD/R recovered the red/green fluorescence ratio, representing a significant increase in mitochondrial membrane potential. The same changing trends in ATP synthesis were observed. We also found that the dynamin-related protein 1 (DRP1), which is a key protein controlling mitochondrial fission,^20^ appearing further increased change in its transcriptional level due to the addition of DADLE (figure 2D). Mitochondria that experience a dynamic cycle of fission and fusion could isolate damaged parts within the mitochondria to maintain their shape, distribution and size.^21^ This evidence suggests that DADLE significantly elevates BMECs’ resistance to OGD/R damage, which might be attributed to the effect of improved mitochondrial function.

### DADLE could elevate the OGD/R-decreased TRPV4-mediated calcium influx in BMECs

**Figure 3:**
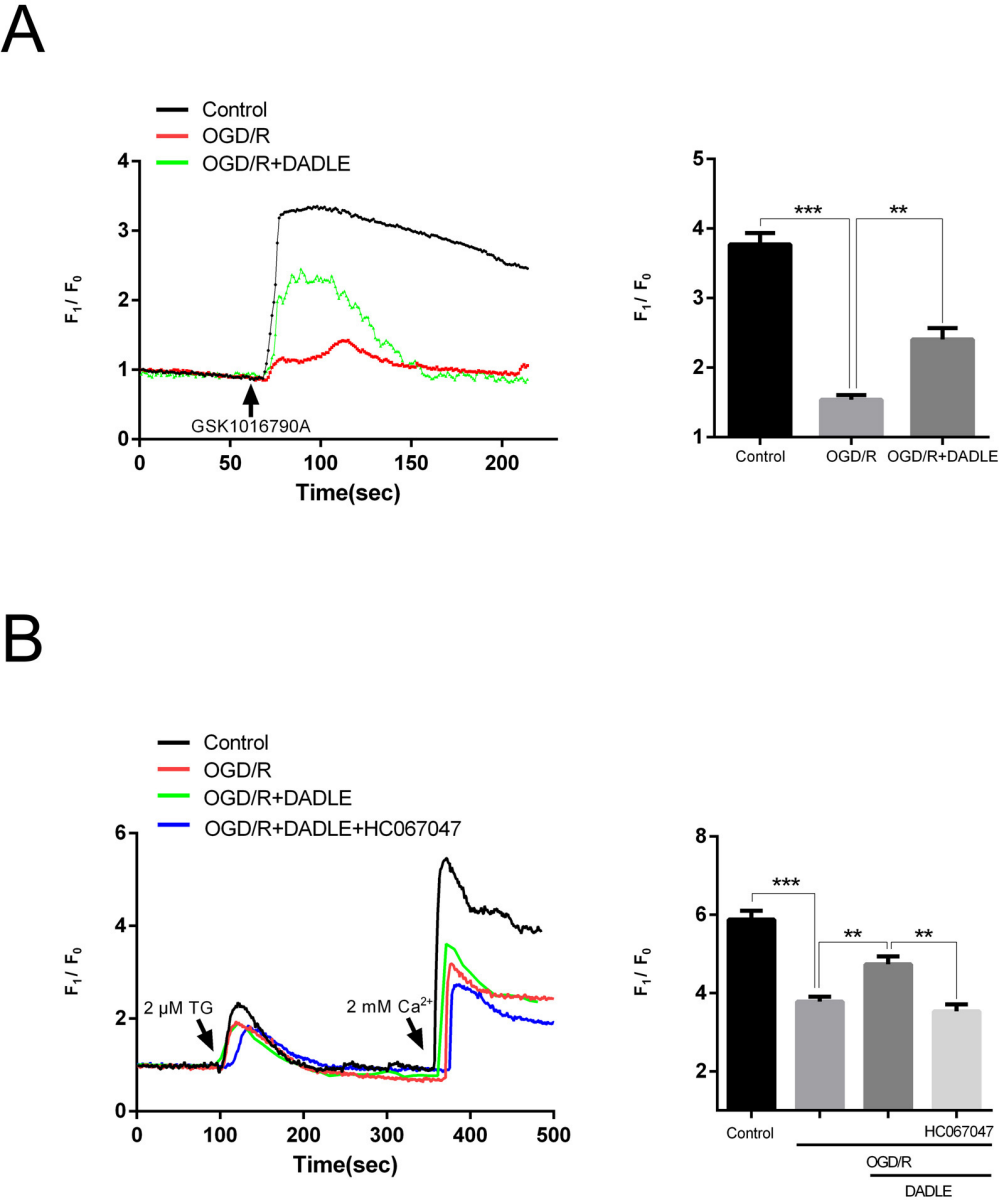
DADLE (5 nM) increases TRPV4-mediated calcium influx in BMECs affected by OGD/R injury. (**A**) Calcium imaging assay was used to detect the signal of calcium ion influx mediated by TRPV4 activated with 50 nM GSK1016790A (n=5). (**B**) In BMECs, after depletion of calcium stores with 2 µM TG, extracellular calcium at 2 mM was provided to detect the signal of SOCE. Concurrent addition of 5 µM HC067047 acted as a TRPV4 inhibitor (n=6). Data were presented as mean±SEM. **p<0.01, ***p<0.001. BMECs, brain microvascular endothelial cells; DADLE, [D-ala2, D-leu5]-Enkephalin; OGD/R, oxygen-glucose deprivation/reoxygenation; SOCE, store-operated Ca^2+^ entry; TRPV4, transient receptor potential subfamily V member 4.

Given that DADLE could both upregulate the expression of TRPV4 and improve mitochondrial function in BMECs, it is speculated that TRPV4 could mediate improvement of mitochondrial function by regulating calcium influx. Hence, we determined whether the calcium influx mediated by GSK1016790A would be affected by OGD/R and DADLE administration. As shown in figure 3A, the OGD/R-damaged cells showed that calcium influx had a weak response to the activation of TRPV4 and that DADLE could rescue it. It is possible that the calcium influx is related to SOCE.^15^ Thus, we attempted to explore whether DADLE treatment influences SOCE and whether it is related to TRPV4. The results (figure 3B) indicate that even after depleting the calcium store with TG followed by exposure to extracellular calcium, there remains a certain calcium influx mediated by SOCE. However, in the OGD/R group—but not the OGD/R+DADLE group—this signal was significantly reduced. If the HC067047 was simultaneously added with DADLE during OGD/R, the enhancement effect of DADLE was blocked. Hence, we hypothesised that DADLE could elevate the OGD/R-decreased TRPV4-mediated calcium influx in BMECs and that it is somewhat correlated with SOCE.

### DADLE could promote the mitophagy of BMECs via upregulated TRPV4

**Figure 4:**
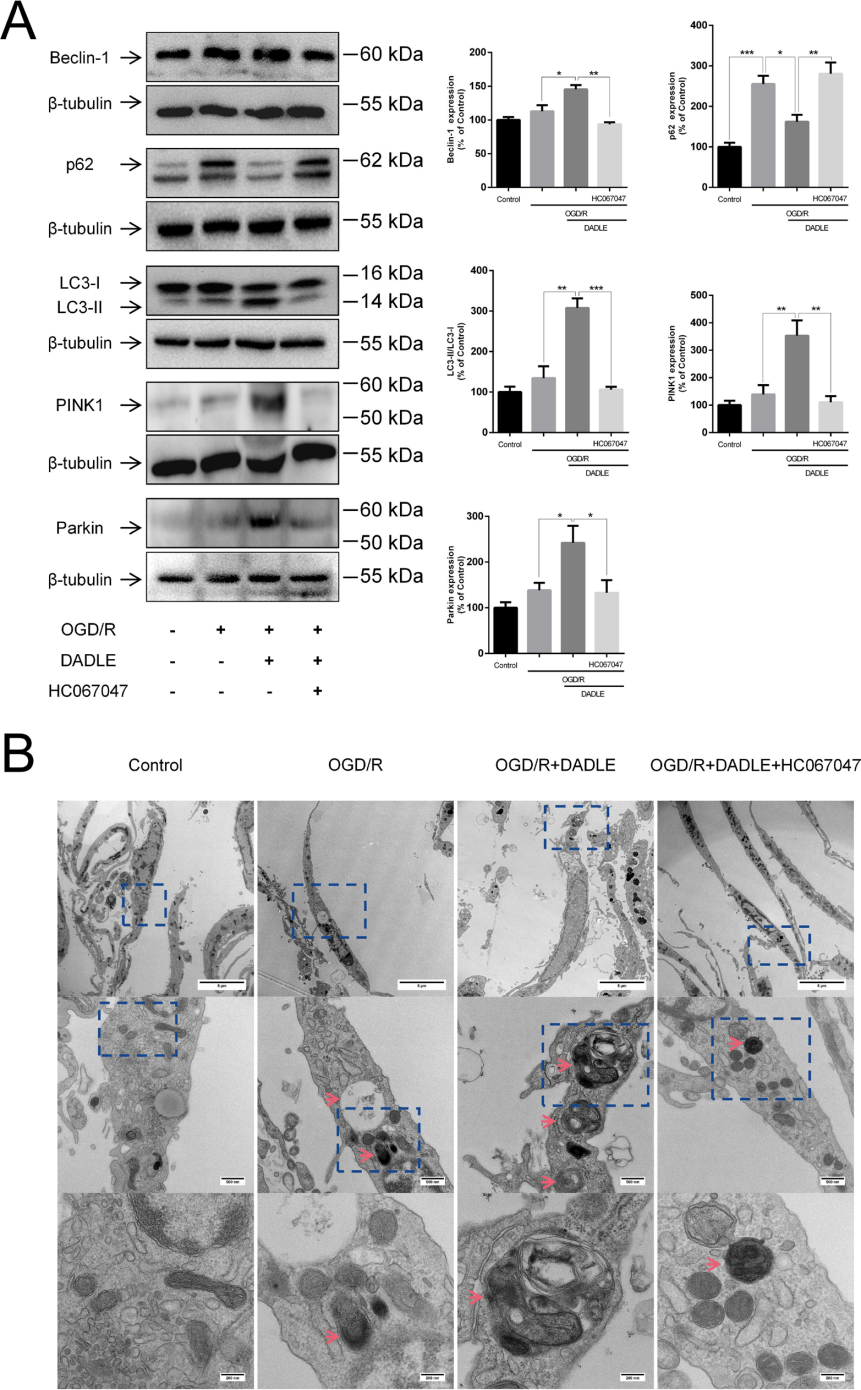
DADLE (5 nM) elevates mitophagy in BMECs under OGD/R via TRPV4. (**A**) Expression levels of autophagy-related proteins Beclin-1 (n=3), p62 (n=8), LC3-II/I (n=3), PINK1 (n=4) and Parkin (n=8) were examined using western blot; 5 µM HC067047 were used to inhibit TRPV4. (**B**) Autophagosome conformation within BMECs was visualised using TEM (n=3). Images taken at increasing magnifications are arranged from top to bottom, with magnifications of 2600×, scale bar=5 µm; 13500×, scale bar=500 nm; 36000×, scale bar=200 nm. Data are presented as mean±SEM. *p<0.05, **p<0.01, ***p<0.001. BMECs, brain microvascular endothelial cells; DADLE, [D-ala2, D-leu5]-Enkephalin; OGD/R, oxygen-glucose deprivation/reoxygenation; TEM, transmission electron microscopy; TRPV4, transient receptor potential subfamily V member 4.

The activation of δORs induces an increase in Ca^2+^ mediated by TRPV4. How does this effect improve mitochondrial function? Noting that calcium signalling is related to mitophagy,^22^ we conjectured that this effect involves the participation of mitophagy, which is a selective process that helps cells envelop and degrade damaged or dysfunctional mitochondria, thus maintaining mitochondrial homeostasis under ischaemic/hypoxic conditions.^23^ First, we detected the expression of autophagy-related proteins in BMECs experiencing OGD/R and found an increase in Beclin-1 and LC3-II/I, indicating the level of cellular autophagy to be increased. DADLE could further promote these trends, whereas HC067047 could block them (figure 4A). The p62 accumulation when OGD/R occurs may suggest the inhibition of autophagic flux within cells due to impaired autophagy.^24^ Therefore, the inhibition of the accumulation in p62 by DADLE might indicate rectified autophagic flux. Additionally, when mitophagy is initiated, it is evidenced by increased protein expression levels of PINK1 and Parkin.^25^ Thus, we further explored whether DADLE could influence those proteins through TRPV4. As shown in figure 4A, the DADLE-triggered amount of PINK1 and Parkin upregulated could be blocked by inhibition of TRPV4. Moreover, using TEM, we observed the location of the appearance of autophagosomes within BMECs (figure 4B). Healthy BMECs displayed almost no autophagic bodies, a small number of autophagosomes were seen in the OGD/R group, and autophagosomes containing folded organelles were observed in the OGD/R group treated with DADLE. This phenomenon was impaired by HC067047. These results confirm that DADLE can enhance autophagy and mitophagy levels in BMECs via TRPV4.

### TRPV4 inhibitor and TRPV4 interference can both suppress the protective effect of the elevated mitochondrial membrane potential, increased ATP content, and improved cell viability induced by DADLE

**Figure 5:**
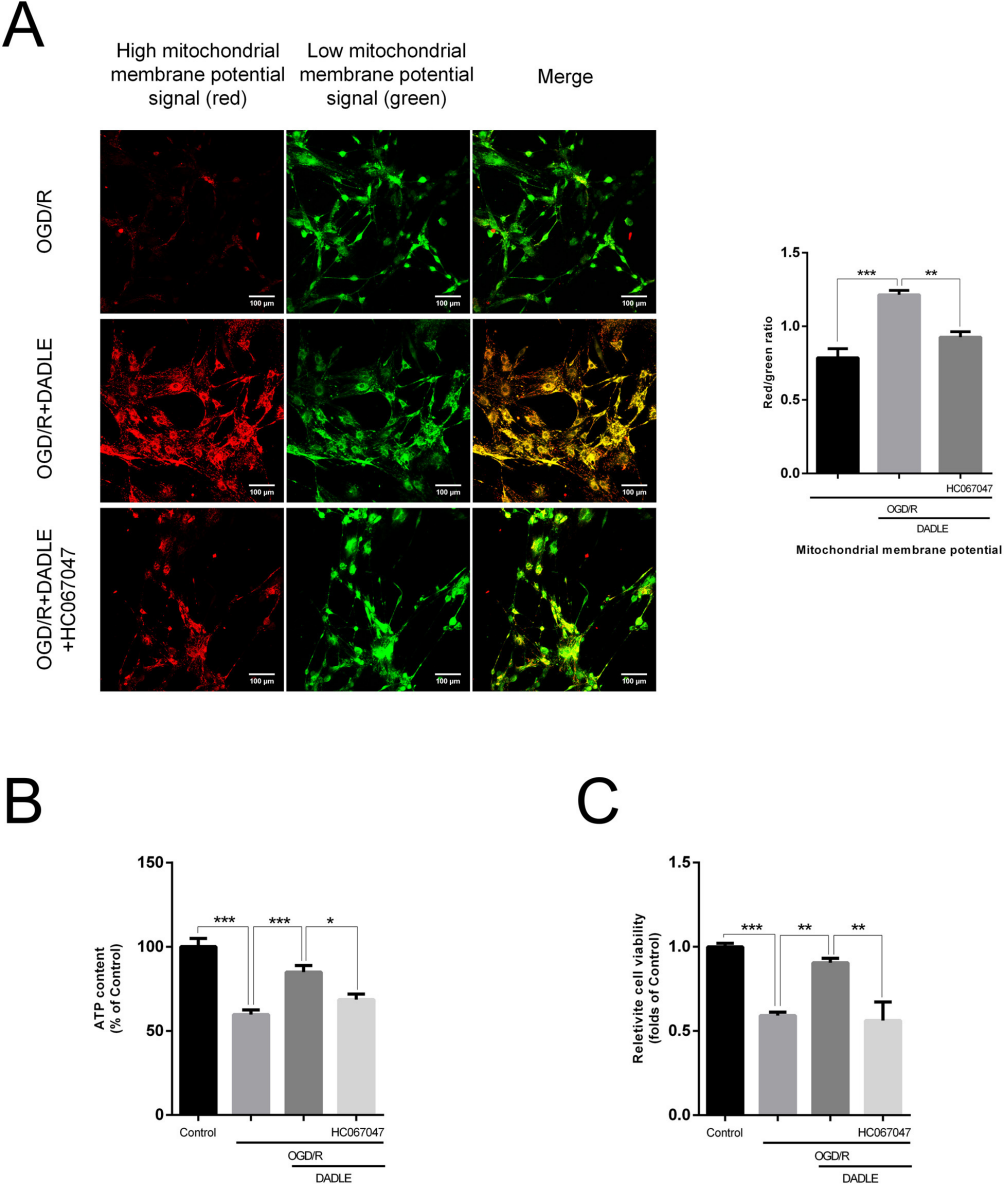
The use of 5 µM HC067047 inhibits the effects of 5 nM DADLE on the mitochondria of BMECs under OGD/R conditions. (**A**) Changes in mitochondrial membrane potential levels following TRPV4 inhibition were evaluated using JC-1 staining under confocal microscopy (n=5). (**B**) ATP synthesis of BMECs after TRPV4 inhibition was examined (n=6). (**C**) Relative cell viability after TRPV4 inhibition was assessed via CCK8 assay (n=6). Data are expressed as mean±SEM. *p<0.05, **p<0.01, ***p<0.001. BMECs, brain microvascular endothelial cells; DADLE, [D-ala2, D-leu5]-Enkephalin; OGD/R, oxygen-glucose deprivation/reoxygenation; TRPV4, transient receptor potential subfamily V member 4.

**Figure 6:**
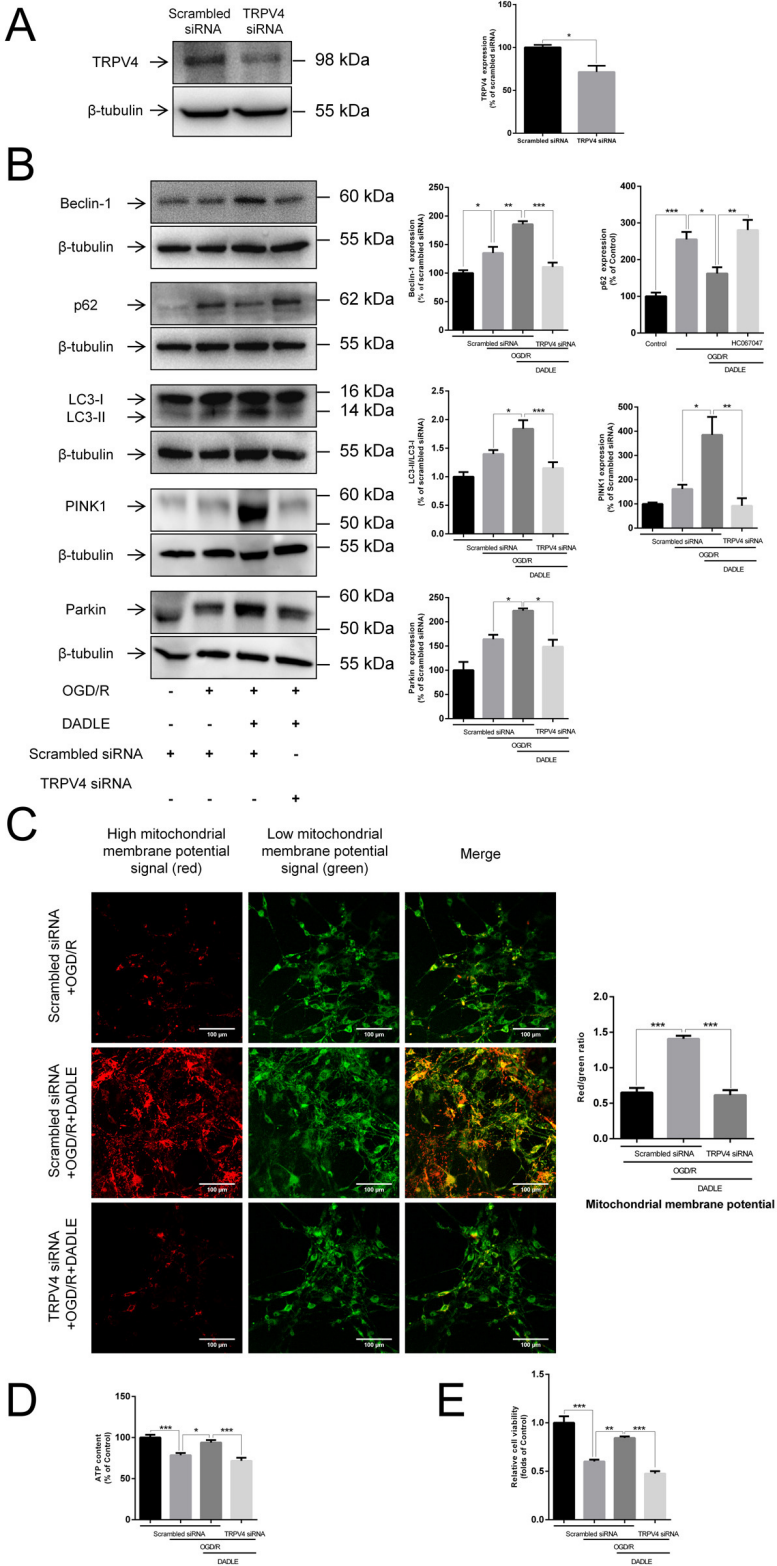
Interference with TRPV4 mRNA synthesis using siRNA. (**A**) The interference effect of siRNA on TRPV4 was measured by western blotting (n=4). (**B**) Determination of the expression of the autophagyrelated proteins Beclin-1 (n=4), p62 (n=7), LC3-II/I (n=6), PINK1 (n=3) and Parkin (n=3) after TRPV4 interference. (**C**) Changes in the level of mitochondrial membrane potential in BMECs after TRPV4 interference (n=9). (**D**) The synthesis of ATP in BMEC cells was examined after TRPV4 interference (n=6). (**E**) Relative cell viability after TRPV4 interference (n=4). Data are expressed as mean±SEM. *p<0.05, **p<0.01, ***p<0.001. BMECs, brain microvascular endothelial cells; DADLE, [D-ala2, D-leu5]-Enkephalin; mRNA, messenger RNA; OGD/R, oxygen-glucose deprivation/reoxygenation; TRPV4, transient receptor potential subfamily V member 4.

Next, we verified the necessity of TRPV4 for the DADLE-exerted protective effect of elevating mitochondrial membrane potential, increasing ATP content and improving cell viability against OGD/R in BMECs. As shown in figures5A  6C, applying the HC067047 and TRPV4 interference can both cause a decline in mitochondrial membrane potential compared with that of cells treated with DADLE, as well as a notable decrease in synthesised ATP amount and a drop in cell vitality (figures5B,C  6D,E). The levels of autophagy-related protein change when TRPV4 expression was knocked down were also consistent with the results of TRPV4 inhibitor application (figure 6B). Meanwhile, we confirmed the presence of an interference effect, as shown in figure 6A. Therefore, it is suggested that TRPV4 mediates the DADLE-induced improvement of mitochondrial function in BMECs subjected to I/R injury.

## Discussion

**Figure 7:**
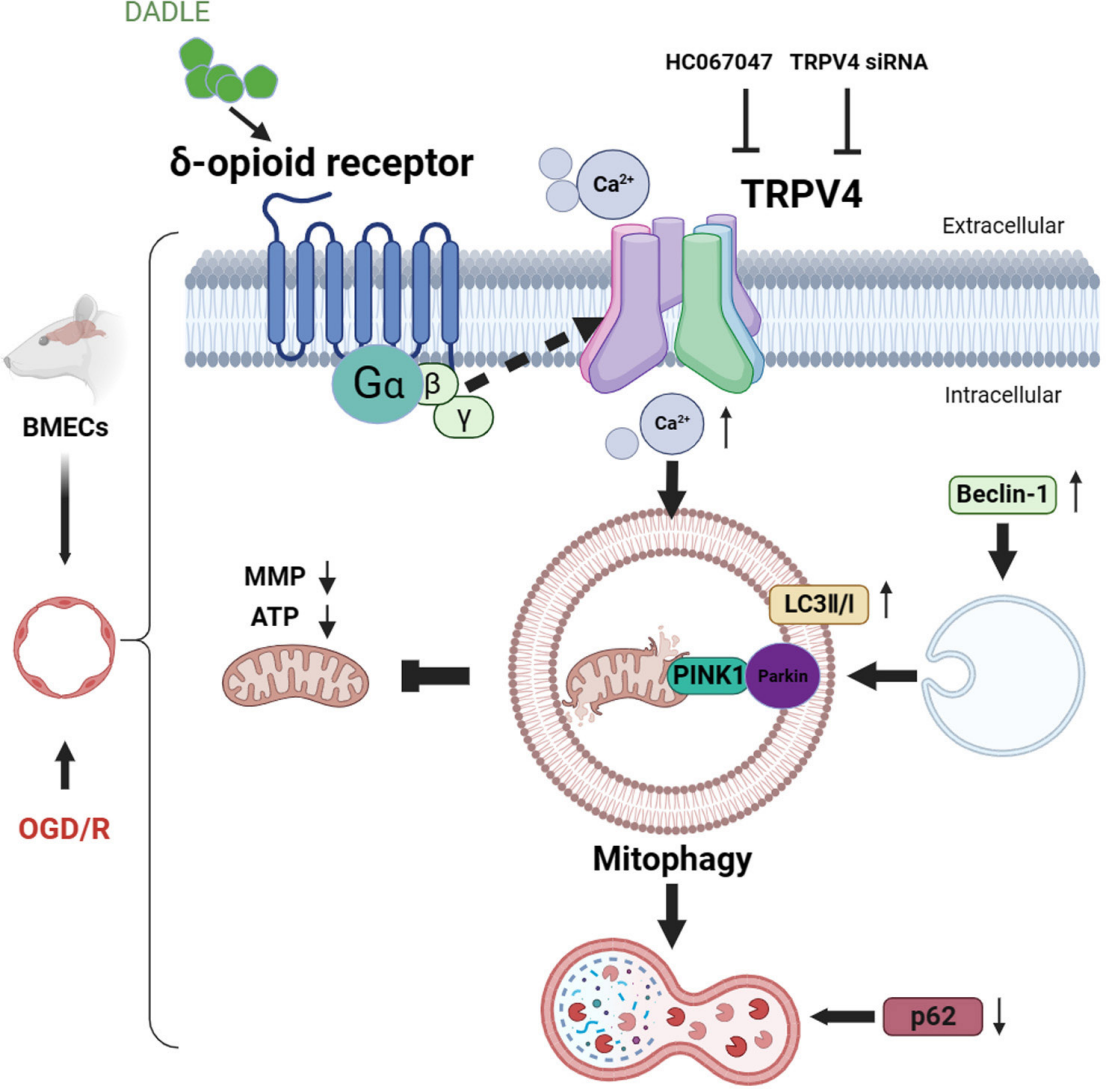
Schematic draw (Created in BioRender: Scientific Image and Illustration Software | BioRender). BMECs were extracted from Sprague-Dawley rats and subjected to the OGD/R model to simulate the I/R process. When the cells were treated with 5 nM DADLE, the expression of TRPV4 protein increased, and TRPV4-mediated calcium influx was enhanced, thereby increasing the level of mitophagy, the mitochondrial membrane potential and ATP synthesis, all while reducing I/R injury and promoting cell survival. The above protective effects of DADLE can be blocked through the addition of a TRPV4 inhibitor (HC067047) or RNAi TRPV4. BMECs, brain microvascular endothelial cells; DADLE, [D-ala2, D-leu5]-Enkephalin; I/R, ischaemia/reperfusion; OGD/R, oxygen-glucose deprivation/reoxygenation; TRPV4, transient receptor potential subfamily V member 4.

In conclusion, the BMECs, when treated with a 5 nM DADLE in vitro model of OGD/R that simulates the I/R process will be upregulated in the expression of TRPV4, and their calcium influx will be mediated by TRPV4 as it intensifies. This, in turn, elevates the level of mitophagy, increases mitochondrial membrane potential, enhances ATP synthesis, alleviates I/R injury and promotes cell survival. The protective effects of DADLE can be obstructed by administering a TRPV4 antagonist or RNAi TRPV4, as shown in figure 7. Based on our previous discovery of endogenous δORs on BMEC cells, in this study, further investigation of the protective mechanism of DADLE-activated δORs against ischaemia/reperfusion injury was elucidated with the proposal of TRPV4 involvement for the first time.

The G protein-coupled receptor (GPCR) and TRP channel families are widely distributed on the cell membrane’s existing crosstalk signalling cascades. PGs, 5-HT and glutamate receptors have been proven to mediate the enhancement of calcium flux through the TRPV1 channel.^26^ In many cases, lipid signals may link the signals from GPCRs to TRPs^27^; for example, arachidonic acid and its metabolites can activate TRPV4.^28^ However, opioid receptors in the GPCR family has not yet been reported to regulate TRP channels.

In terms of physiological function, TRP channels are suitable for drug targets of pain.^29^ The analgesic effect of δOR agonists (eg, DADLE) has long been validated.^30^ Although this study was not conducted in the context of pain, it is the first to discover that δOR activated by DADLE can regulate the expression of TRPV4 and control the mitophagy pathway under I/R conditions.

As to how δOR regulates TRPV4 signals, on one hand, downstream δOR can activate the MAPK and Akt signalling pathways^31^ or possibly cause PIP2 decomposition, thereby regulating TRP channel proteins.^32^ Recent studies have found that the G protein subunit Gβγ can loosely attach to TRPM3 to form a complex.^33^ Protein kinase C is also possibly one of the activated downstream kinases of δOR that regulate TRP channels.^34^ On the other hand, we can identify molecules that can activate TRP channels as well as take part in how δOR is commonly signalled. For example, the first endogenous agonist of TRPV1 discovered was endogenous cannabinoid anandamide (AEA).^35^ This endogenous cannabinoid system interacts with opioids in terms of antinociception.^36^

In recent years, research on δOR agonists as tissue protectants under I/R conditions have continuously been published, yet its specific mechanisms remain unclear. Zhao *et al* reported that δOR agonists may develop antihypoxic-ischaemic effects by elevating the levels of cellular autophagy.^37^ This cardioprotective effect also involves an increase in mitochondrial membrane potential, which is consistent with our findings. Through differentially expressed mRNA screening, we found TRPV4 participated in this process. Given that TRPV4 is a cation ion channel and that calcium ions may engage in autophagy activation under stress conditions,^38^ we further explored whether calcium influx through TRPV4 may regulate mitophagy levels and improve mitochondrial function. A similar mechanism is also evident in terms of the key role of calcium flux induced by TRPV4 activation in the initiation of mitophagy by cannabidiol^39^; however, this effect was discovered to cause glioma cell death rather than promote survival. Specifically, whether the downstream of δOR directly affects the calcium influx mediated by TRPV4 or indirectly changes the SOCE-induced calcium influx resulting from TRPV4 is still unclear because both mechanisms seem to be involved (figure 3A, B).

Direct regulation of the TRPV4 ion channel may become a therapeutic approach for ischaemic stroke. It has been reported that TRPV4 activation by 4α-phorbol 12,13-didecanoate (4α-PDD) in rats promotes the functional recovery from ischaemic stroke by promoting angiogenesis and neurogenesis.^11^ Although opioids have issues of addiction and tolerance, considering their analgesic and antidepressant properties, the rational application of opioids in the treatment or preconditioning of hypoxia-ischaemia may produce multiple benefits. In the current study, we did not separate the extraction of male and female rats’ brains for the respective BMECs culture. The next work could focus on the sex difference influence of the δ opioid peptides on the injury of I/R. In summary, the tissue-protective mechanism proposed in this study can provide a theoretical basis for exploring new therapies and drug targets.

## supplementary material

10.1136/svn-2023-003080 online supplemental figure 1

10.1136/svn-2023-003080 online supplemental material 1

## Data Availability

Data are available upon reasonable request.
