# Supplementary figures and images for: Delta opioid peptide [D-ala2, D-leu5]-Enkephalin’s ability to enhance mitophagy via TRPV4 to relieve ischemia/reperfusion injury in brain microvascular endothelial cells
Source: Stroke Vasc Neurol. 2024 May 2;10(1):e003080. doi: 10.1136/svn-2023-003080 (PMC11877439; doi:10.1136/svn-2023-003080)

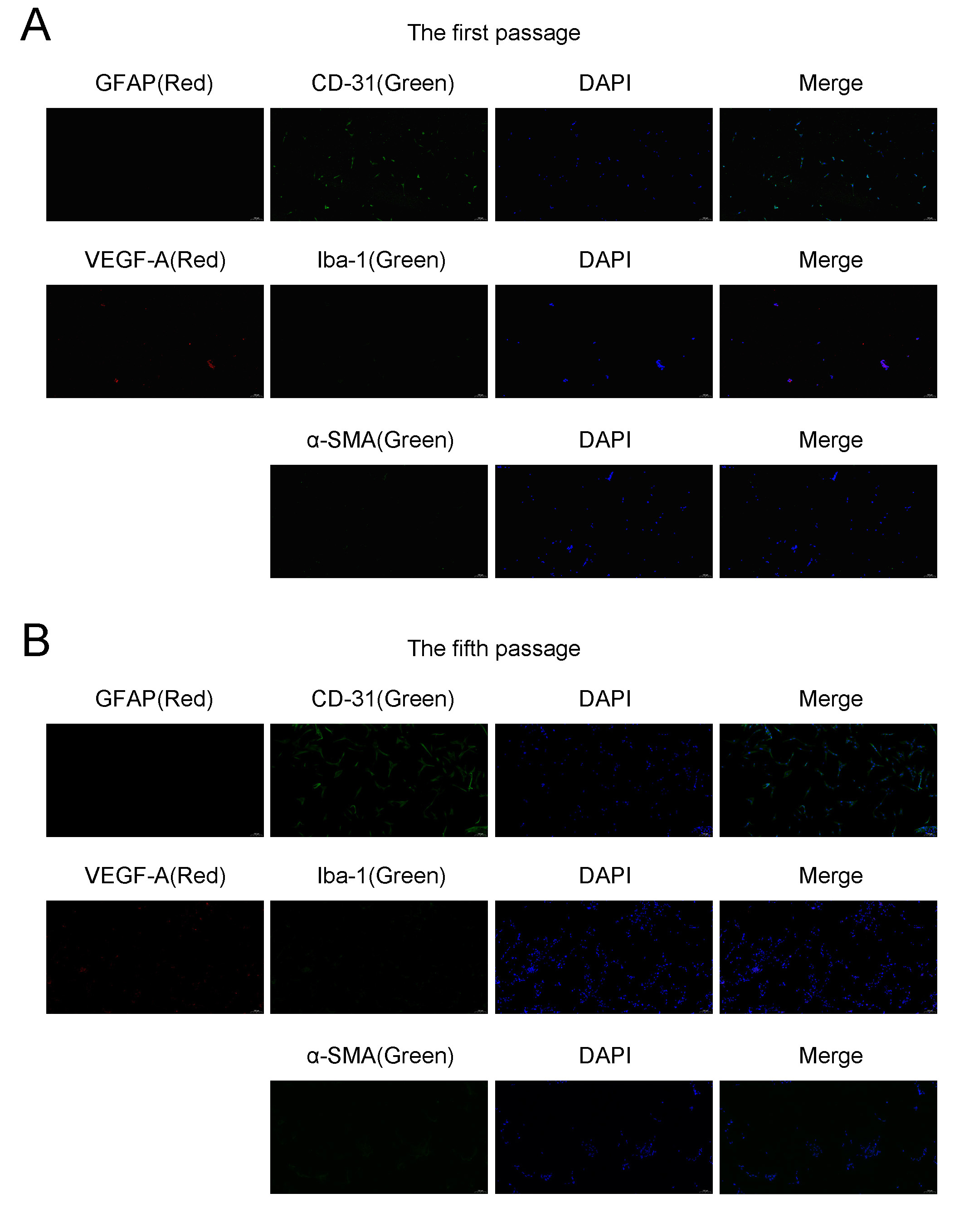

Supplement: online supplemental figure 1 [file svn-10-1-s001.jpg]
