# Supplementary material for: Delta opioid peptide [D-ala2, D-leu5]-Enkephalin’s ability to enhance mitophagy via TRPV4 to relieve ischemia/reperfusion injury in brain microvascular endothelial cells
Source: Stroke Vasc Neurol. 2024 May 2;10(1):e003080. doi: 10.1136/svn-2023-003080 (PMC11877439; doi:10.1136/svn-2023-003080)

Original wester blot for Figure 1E

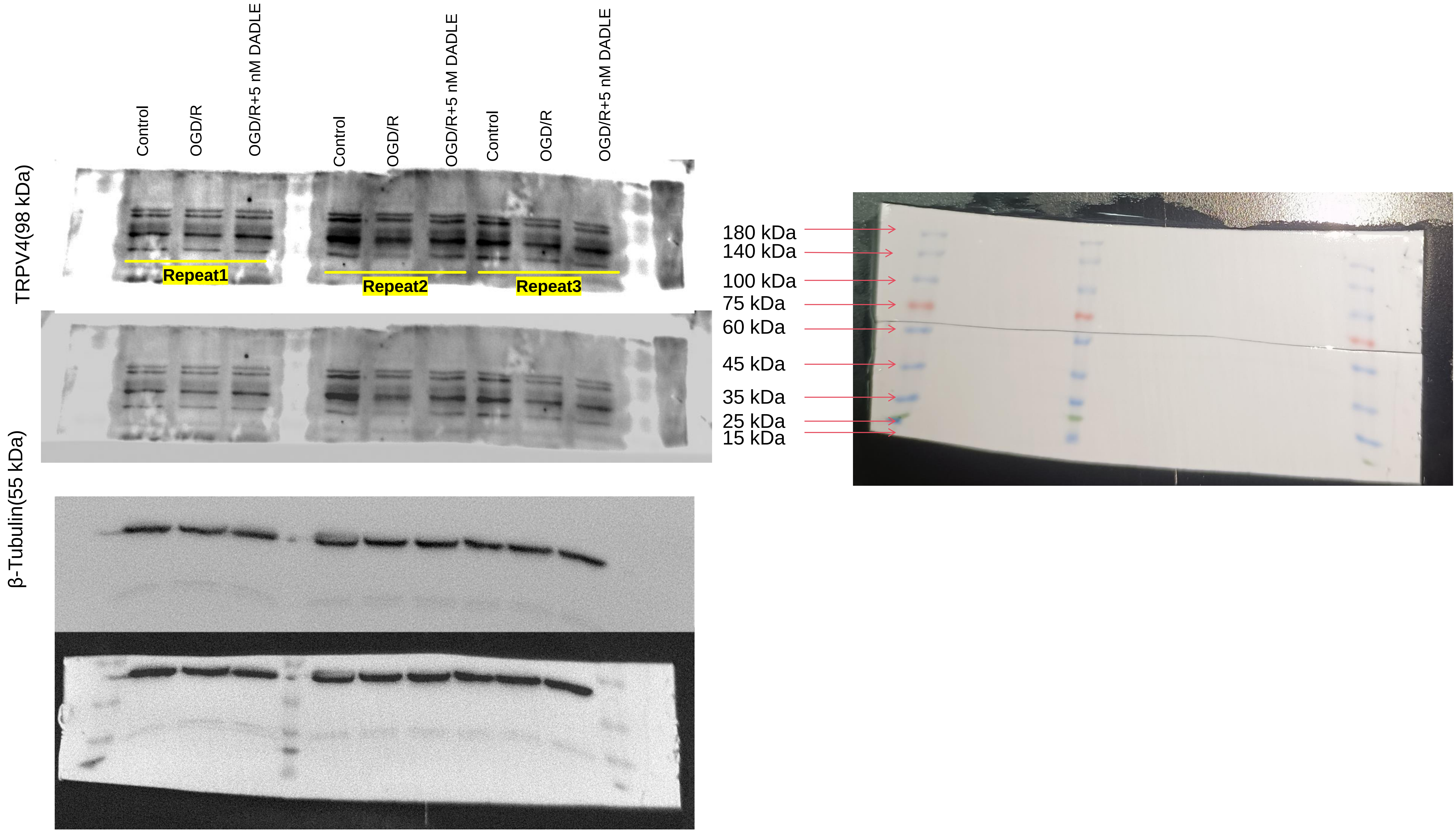

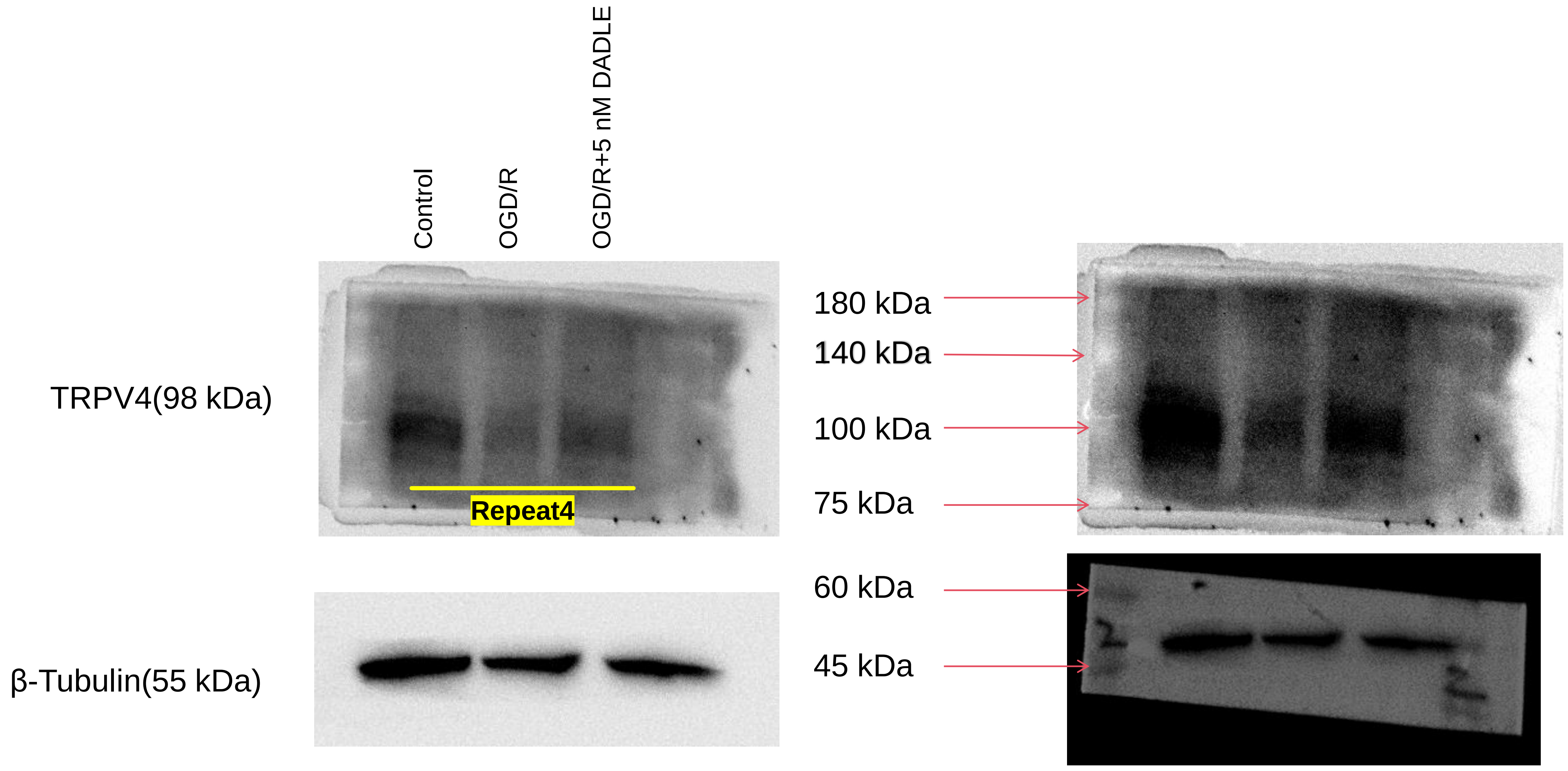

Original wester blot for Figure 1F

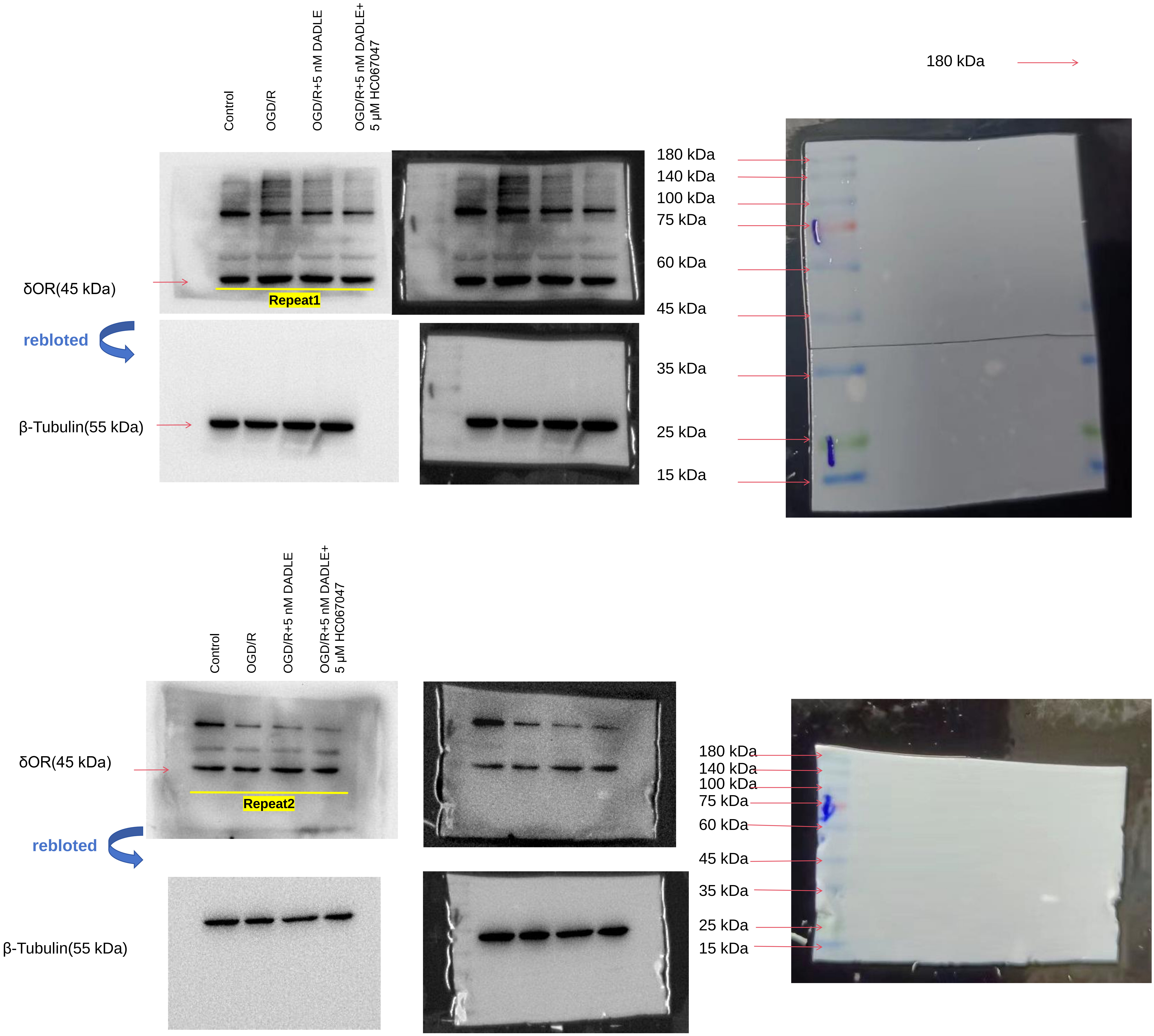

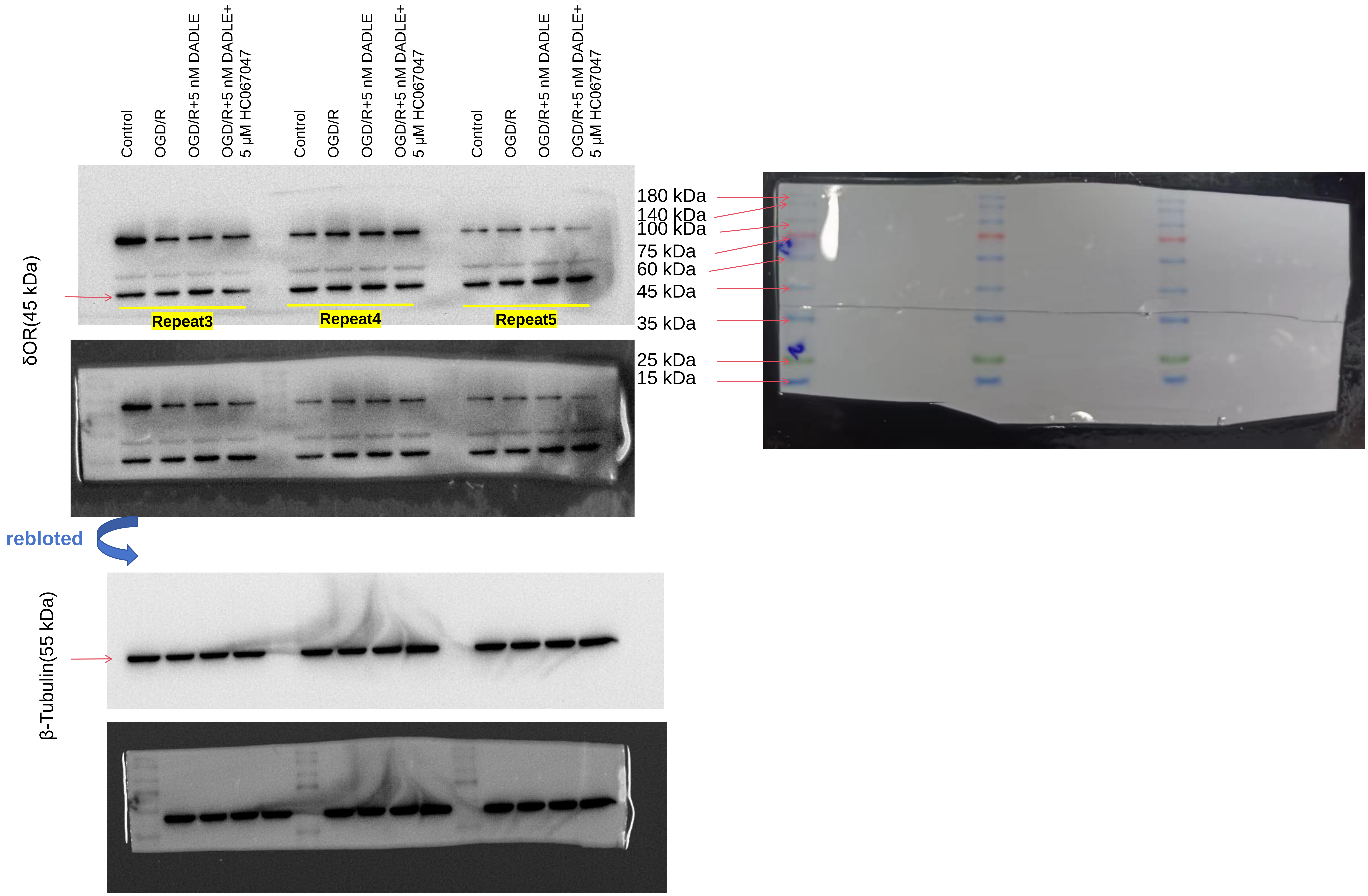

Original wester blot for Figure 4A(Beclin-1)

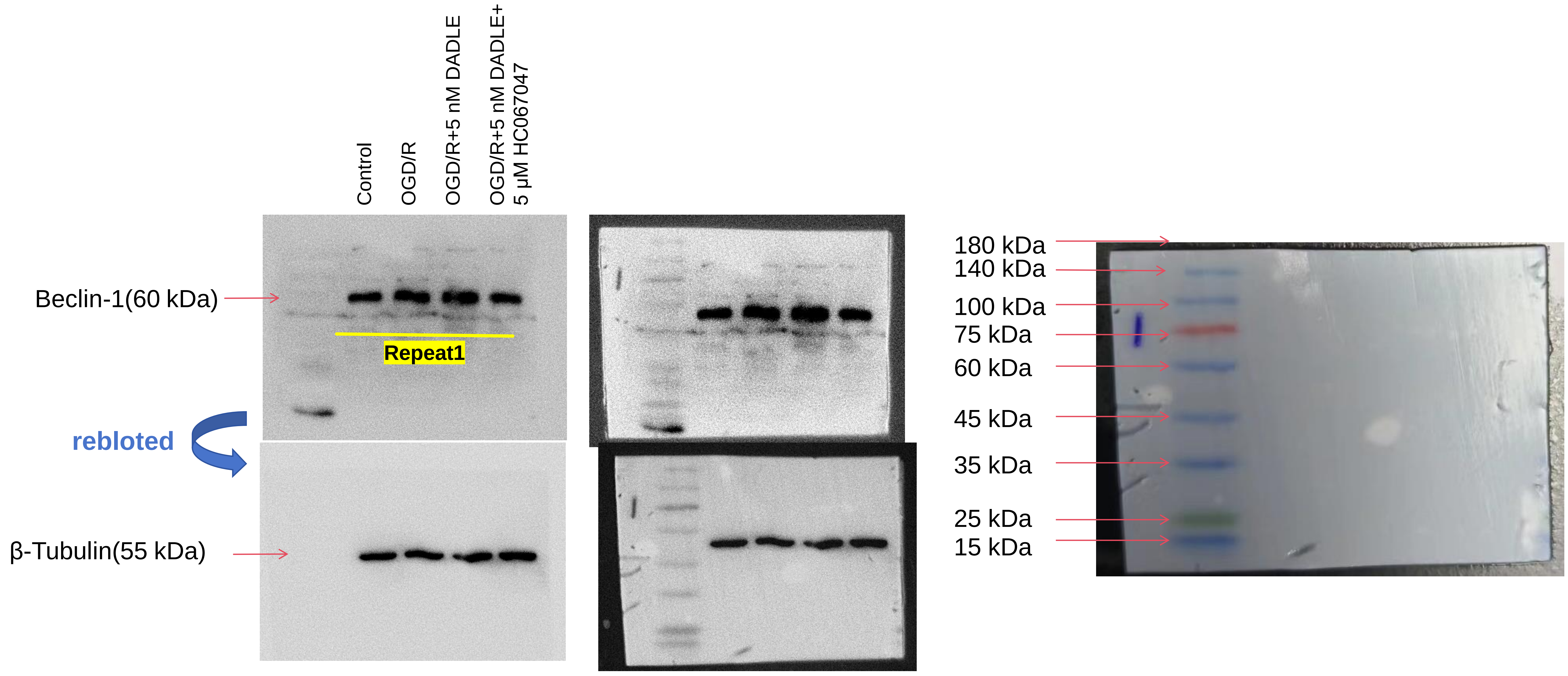

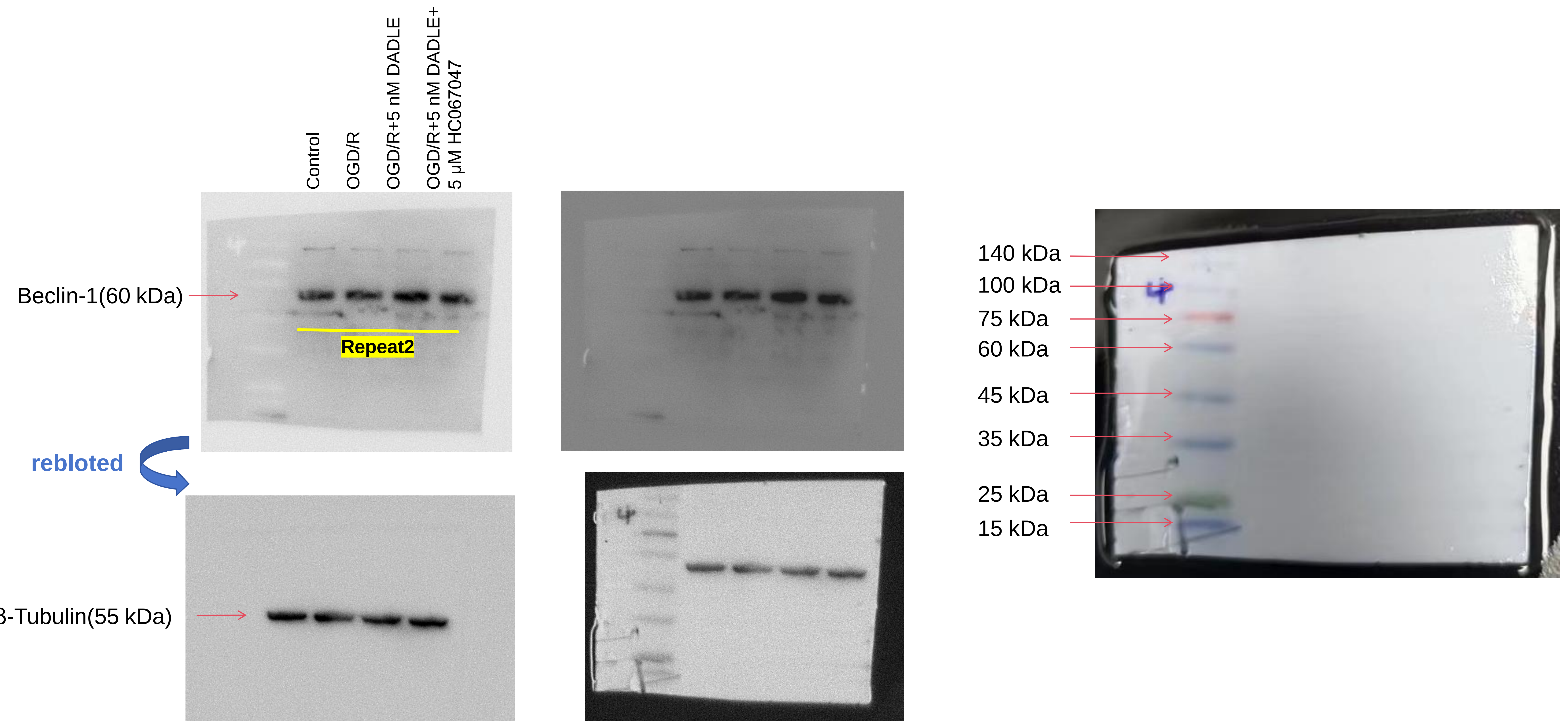
Control
OGD/R
OGD/R+5 nM DADLE
OGD/R+5 nM DADLE+
5 μM HC067047
Beclin-1(60 kDa)
Repeat2
rebloted
-Tubulin(55 kDa)
140 kDa
100 kDa
75 kDa
60 kDa
45 kDa
35 kDa
25 kDa
15 kDa

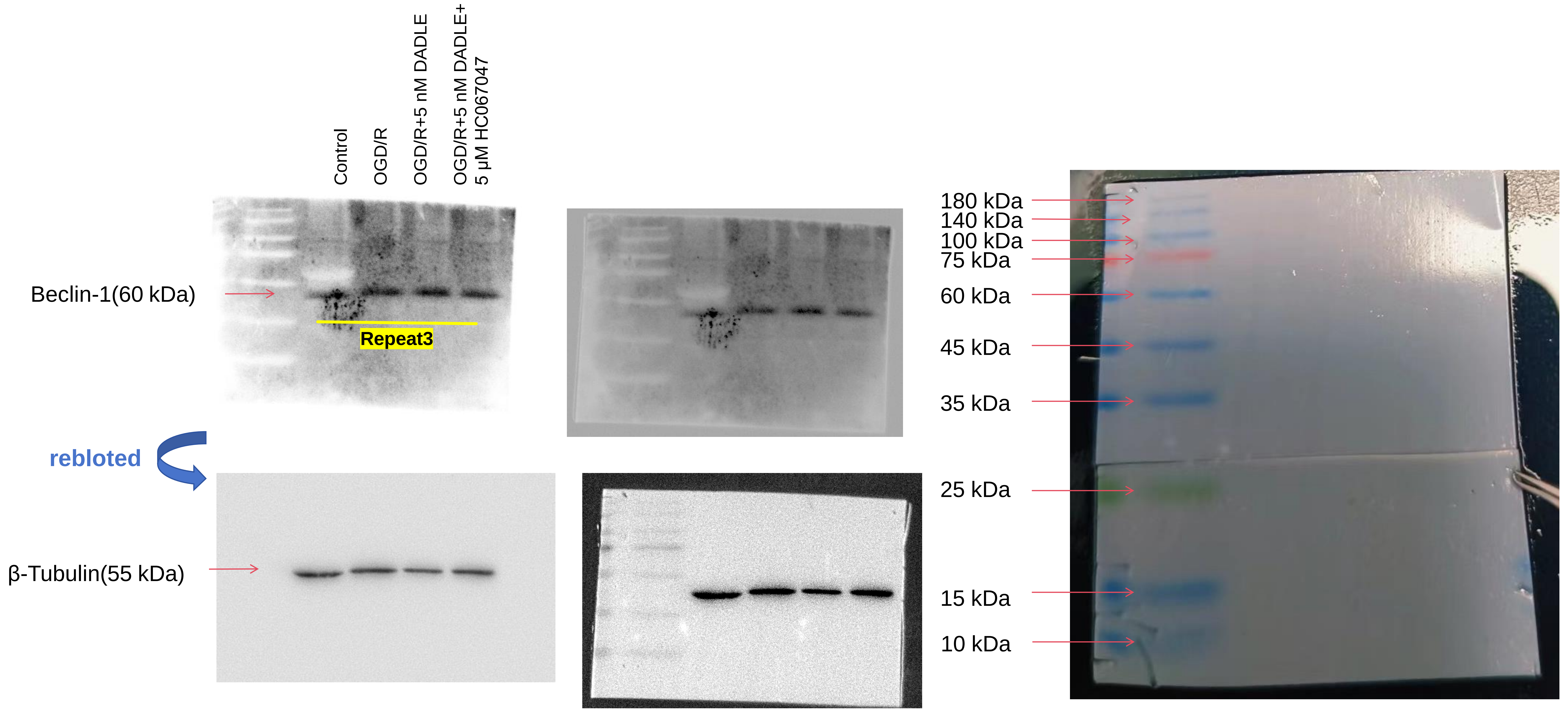
Control
OGD/R
OGD/R+5 nM DADLE
OGD/R+5 nM DADLE+
5 μM HC067047
Beclin-1(60 kDa)
Repeat3
rebloted
β-Tubulin(55 kDa)
180 kDa
140 kDa
100 kDa
75 kDa
60 kDa
45 kDa
35 kDa
25 kDa
15 kDa
10 kDa

Original wester blot for Figure 4A(p62)

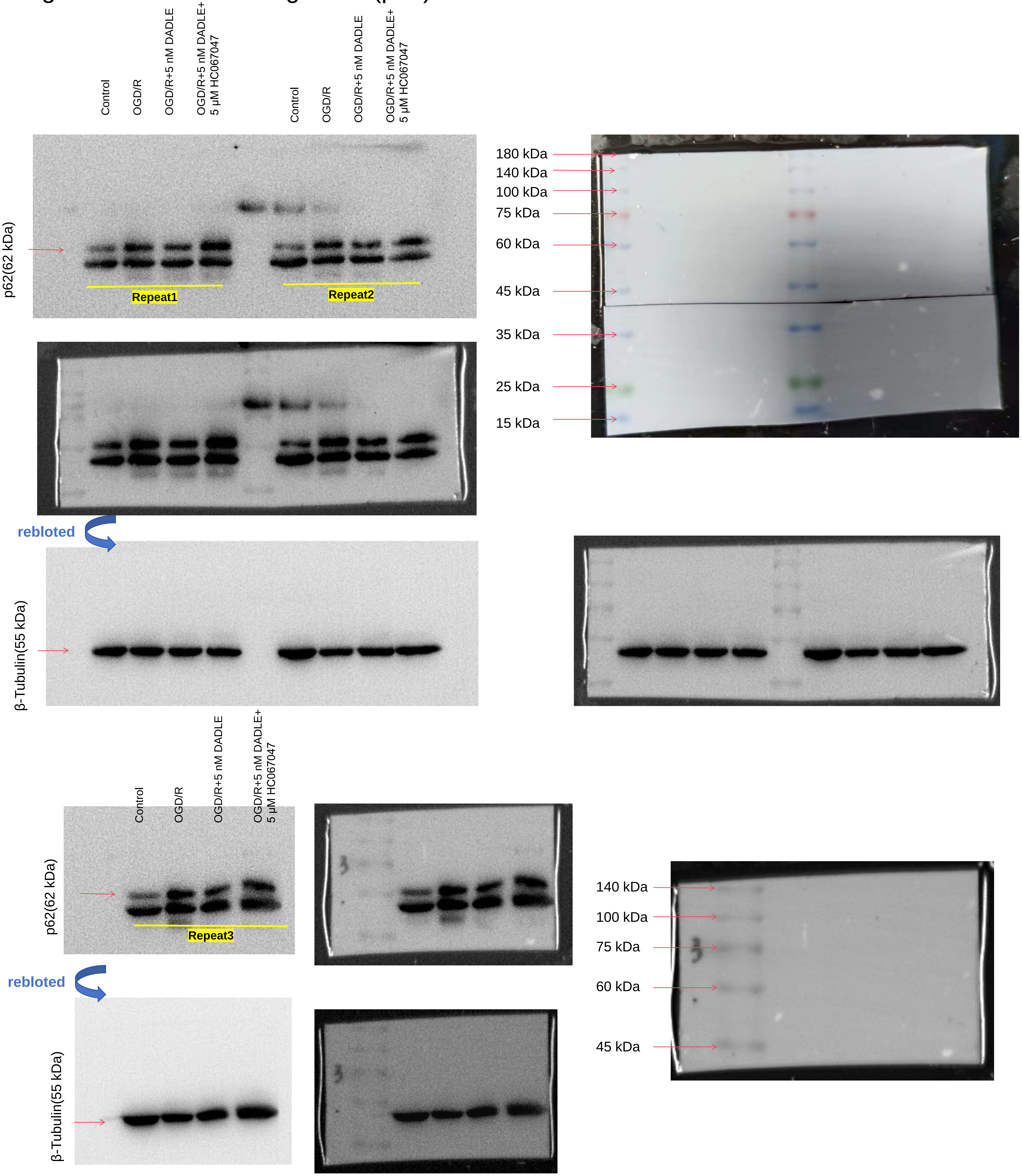

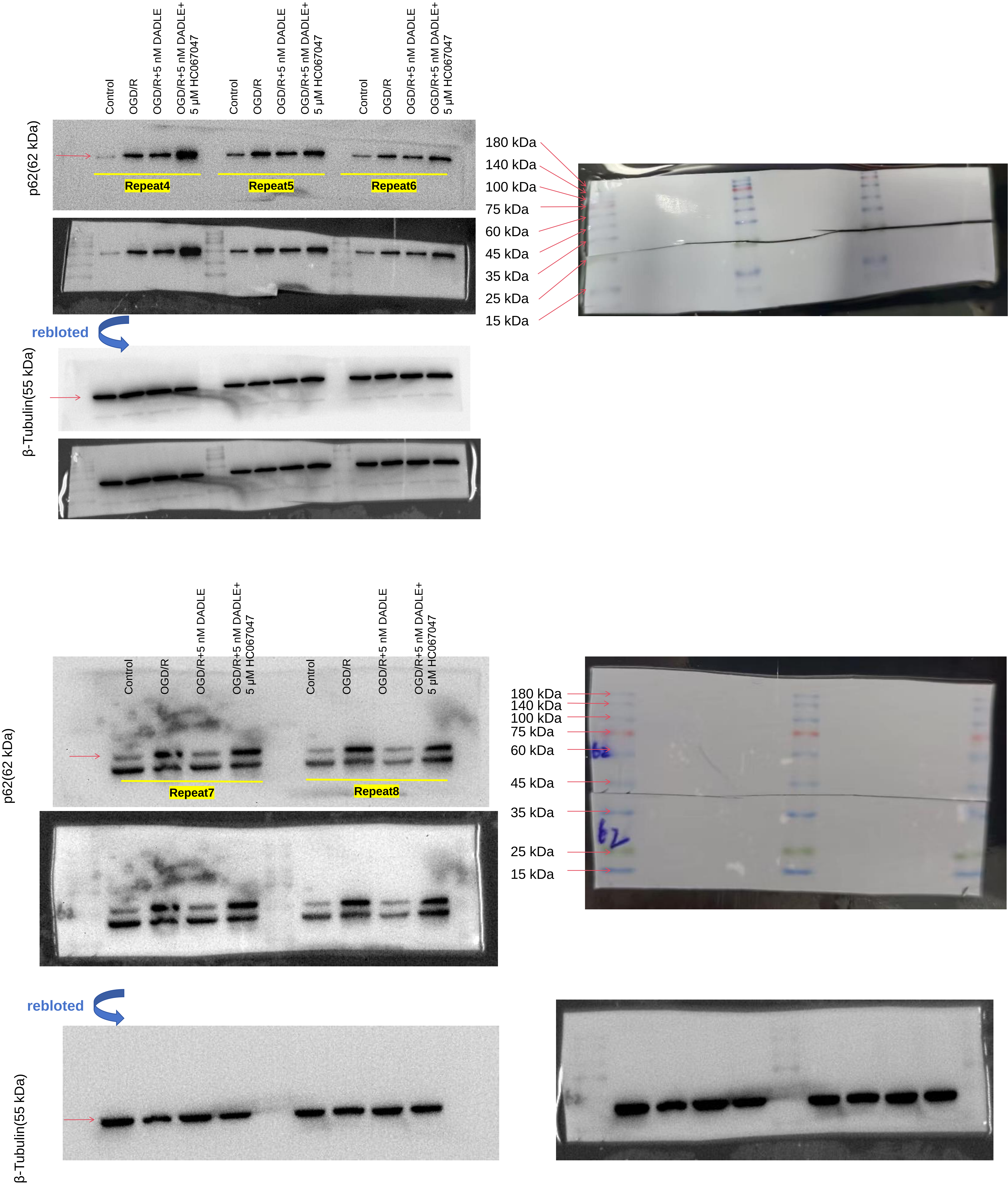

Control
OGD/R
OGD/R+5 nM DADLE
OGD/R+5 nM DADLE+
5 μM HC067047
p62(62 kDa)
Repeat4
Repeat5
Repeat6
180 kDa
140 kDa
100 kDa
75 kDa
60 kDa
45 kDa
35 kDa
25 kDa
15 kDa
rebloted
β-Tubulin(55 kDa)
Control
OGD/R
OGD/R+5 nM DADLE
OGD/R+5 nM DADLE+
5 μM HC067047
p62(62 kDa)
Repeat7
Repeat8
180 kDa
140 kDa
100 kDa
75 kDa
60 kDa
45 kDa
35 kDa
25 kDa
15 kDa
rebloted
β-Tubulin(55 kDa)

Original wester blot for Figure 4A(LC3Ⅱ、Ⅰ)

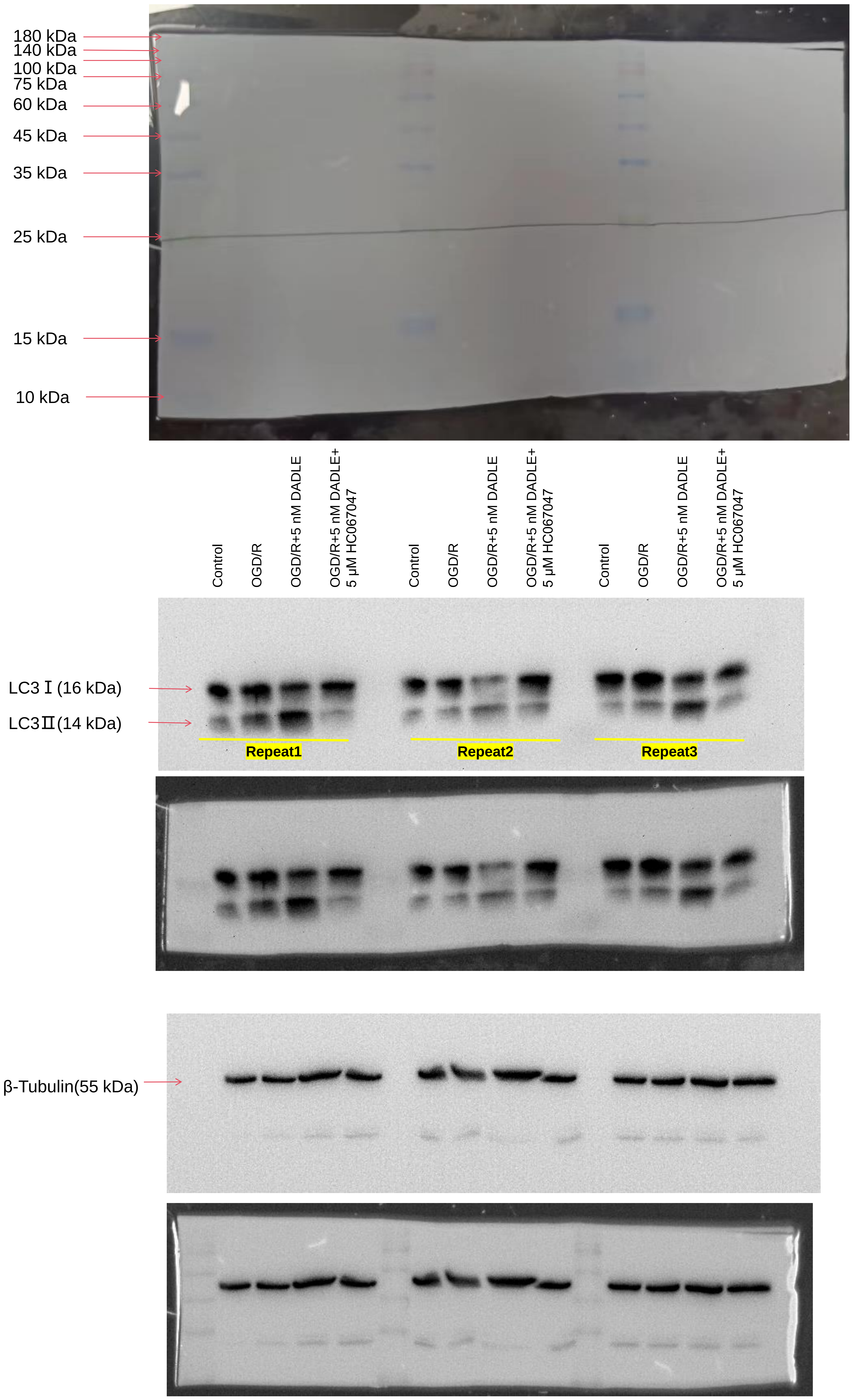

Original wester blot for Figure 4A(PINK1)

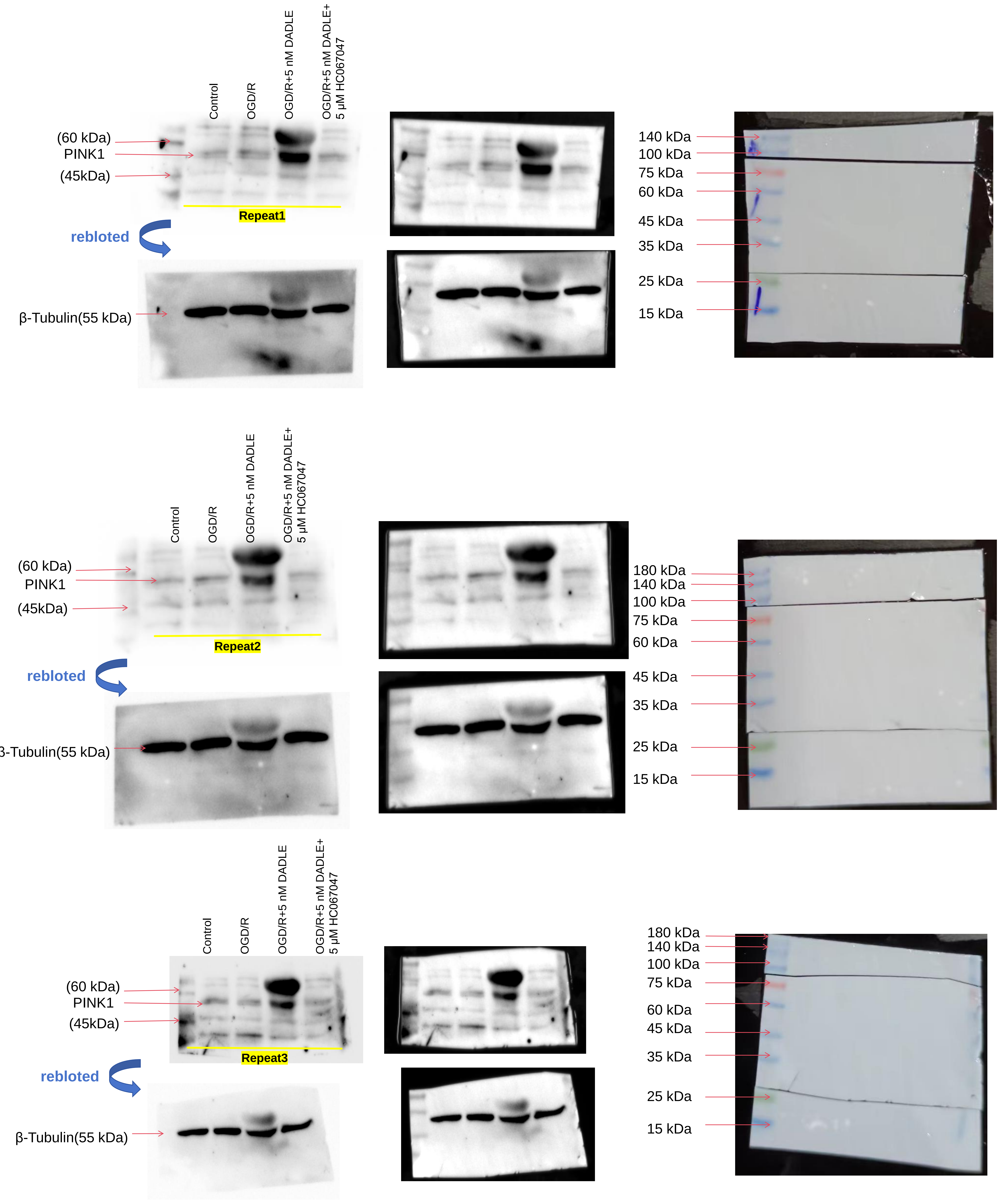

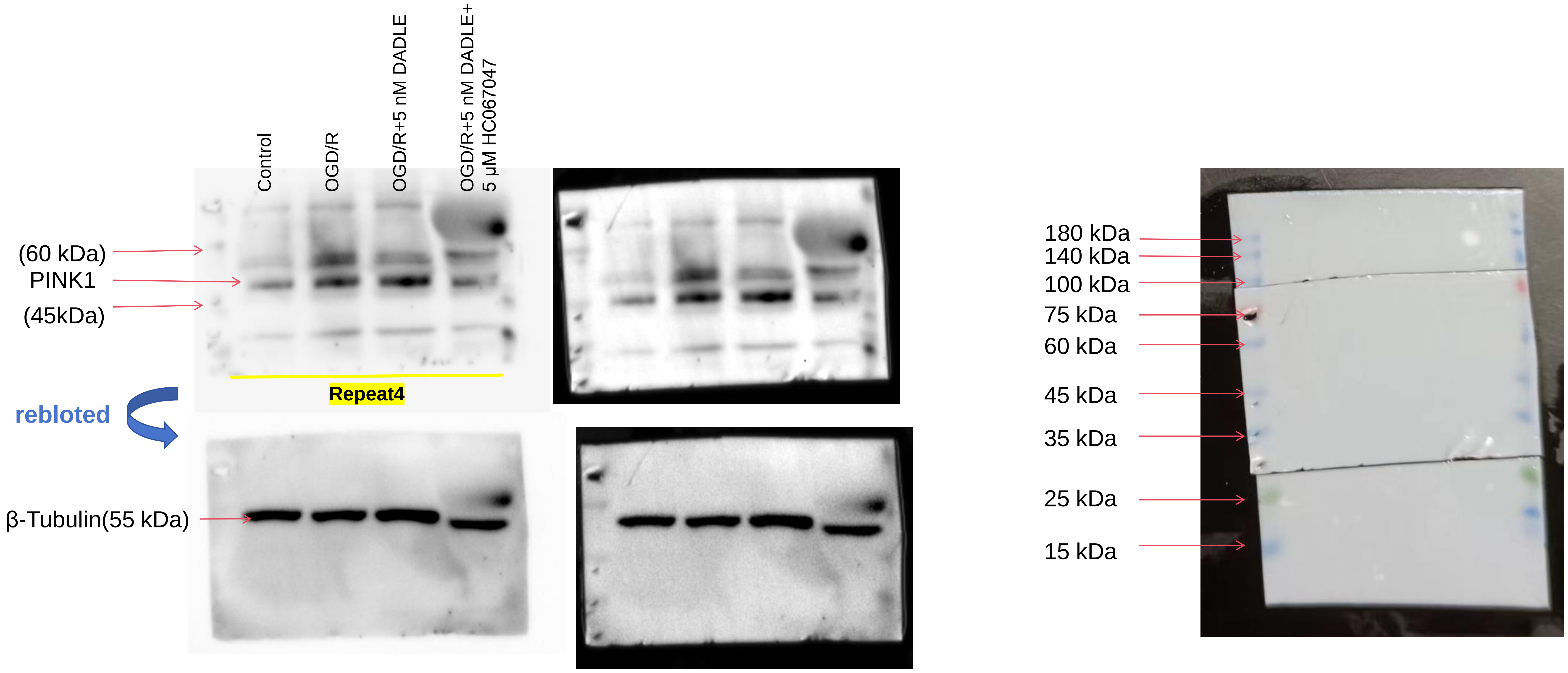

Original wester blot for Figure 4A(Parkin)

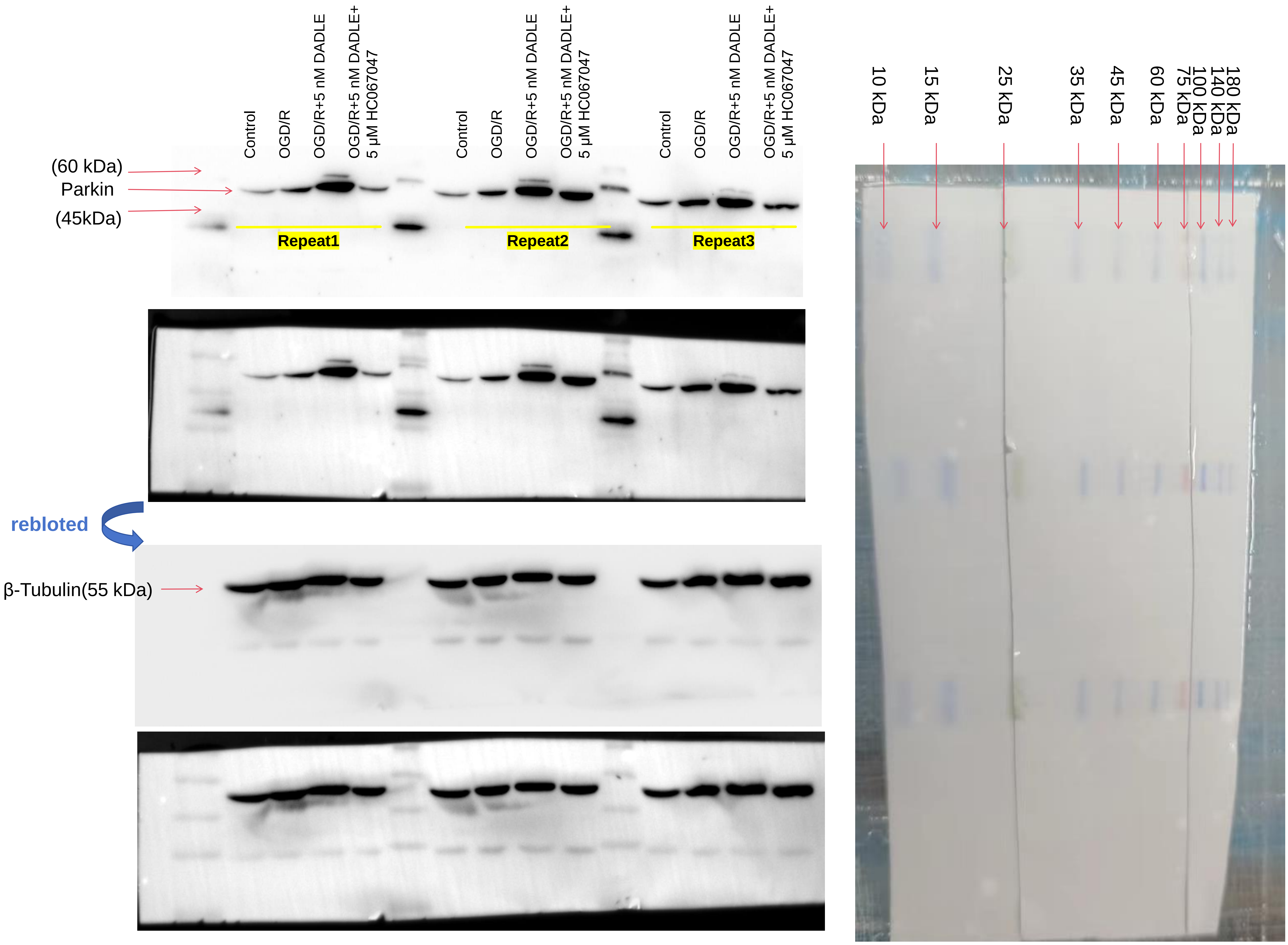

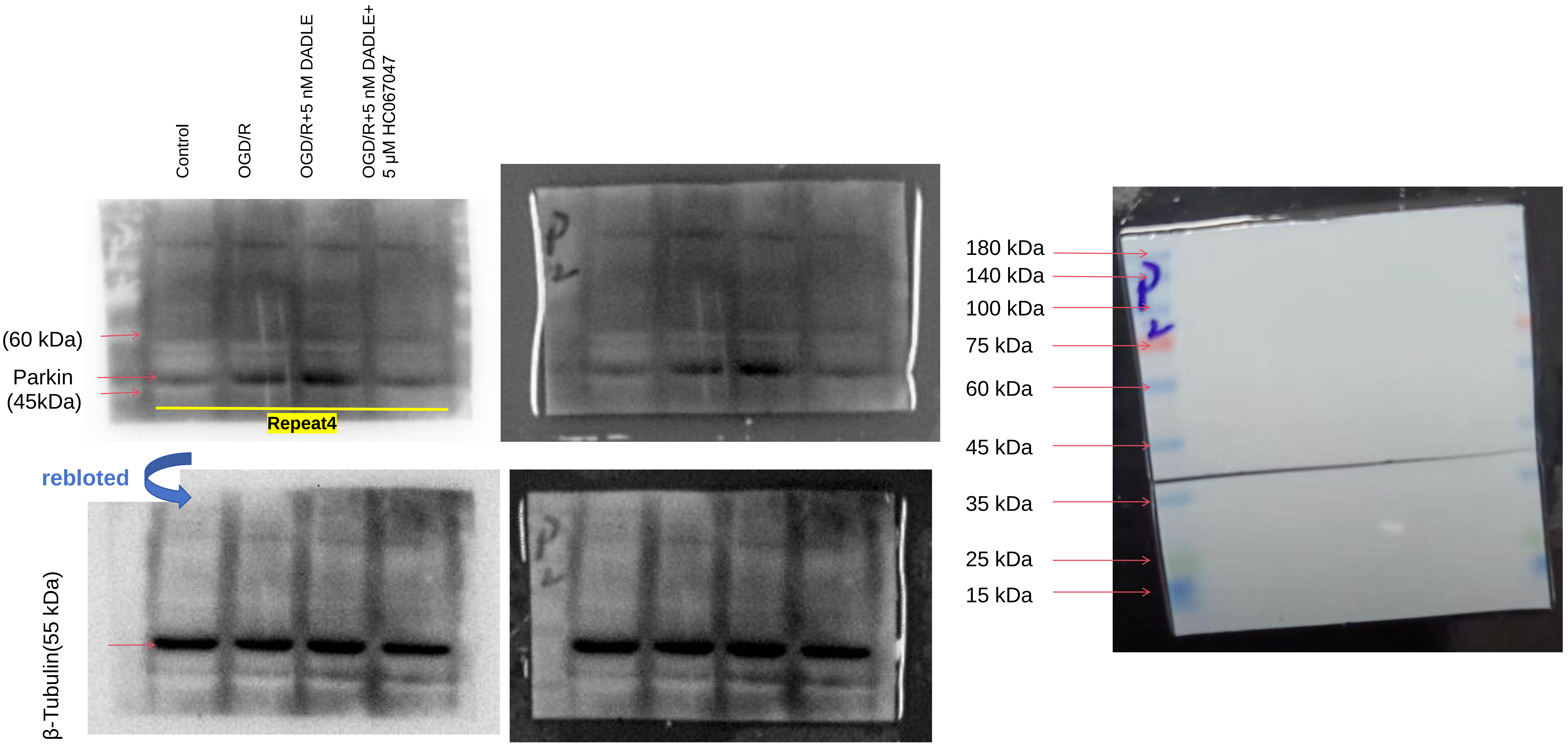

Control
OGD/R
OGD/R+5 nM DADLE
OGD/R+5 nM DADLE+
5 μM HC067047
(60 kDa)
Parkin
(45kDa)
Repeat4
rebloted
β-Tubulin(55 kDa)
180 kDa
140 kDa
100 kDa
75 kDa
60 kDa
45 kDa
35 kDa
25 kDa
15 kDa

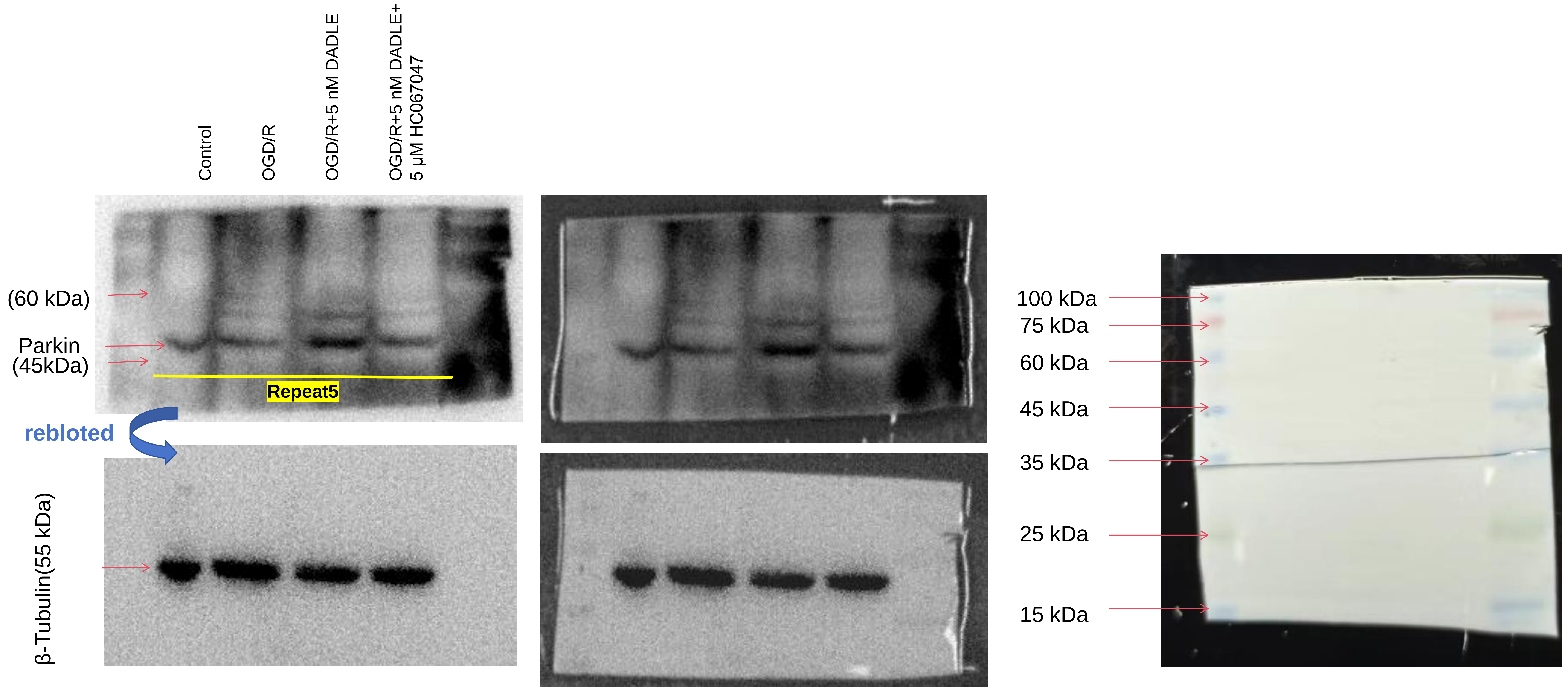

Control
OGD/R
OGD/R+5 nM DADLE
OGD/R+5 nM DADLE+
5 μM HC067047
(60 kDa)
Parkin
(45kDa)
Repeat5
rebloted
β-Tubulin(55 kDa)
100 kDa
75 kDa
60 kDa
45 kDa
35 kDa
25 kDa
15 kDa

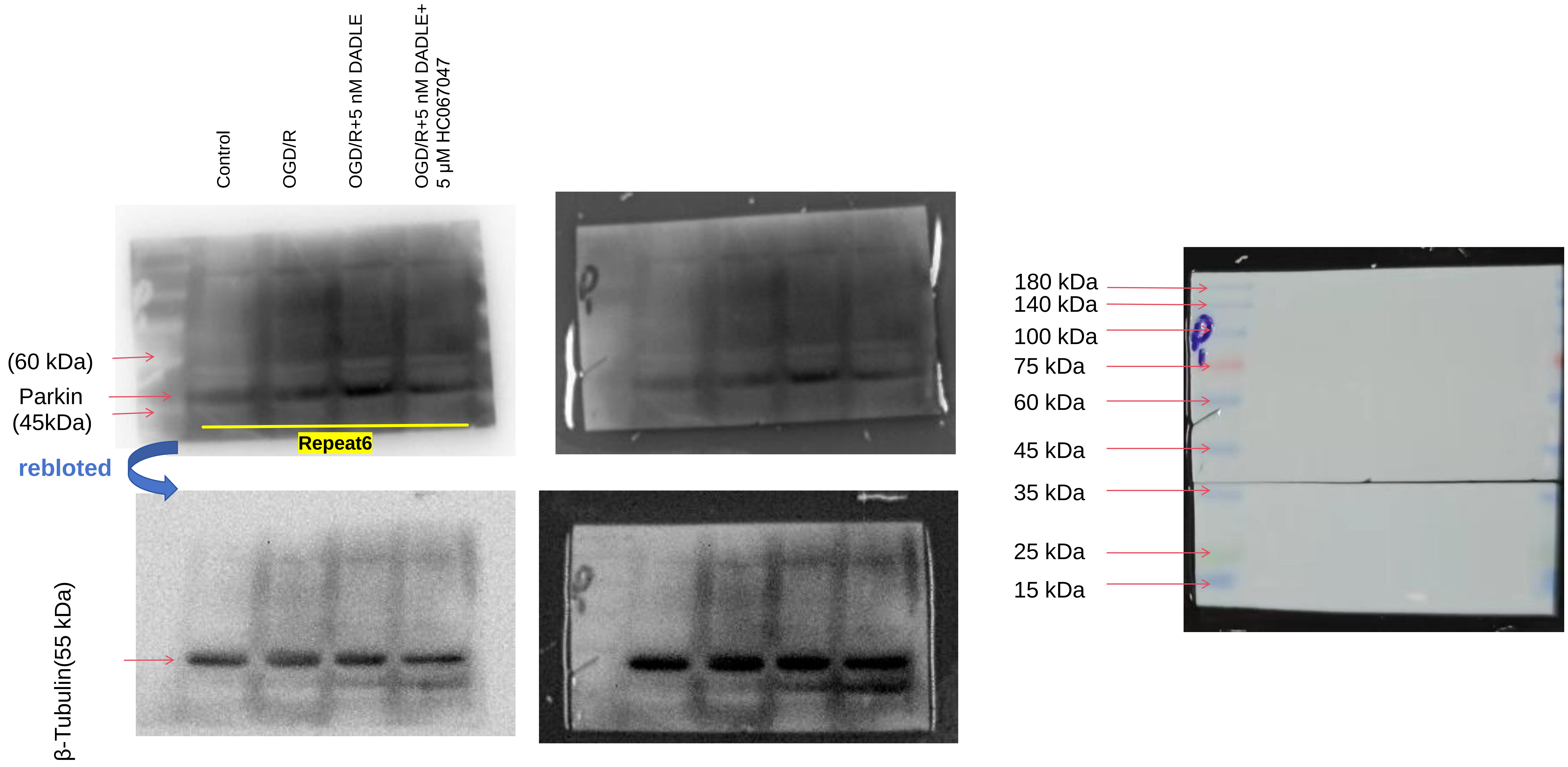
Control
OGD/R
OGD/R+5 nM DADLE
OGD/R+5 nM DADLE+
5 μM HC067047
(60 kDa)
Parkin
(45kDa)
Repeat6
rebloted
β-Tubulin(55 kDa)
180 kDa
140 kDa
100 kDa
75 kDa
60 kDa
45 kDa
35 kDa
25 kDa
15 kDa

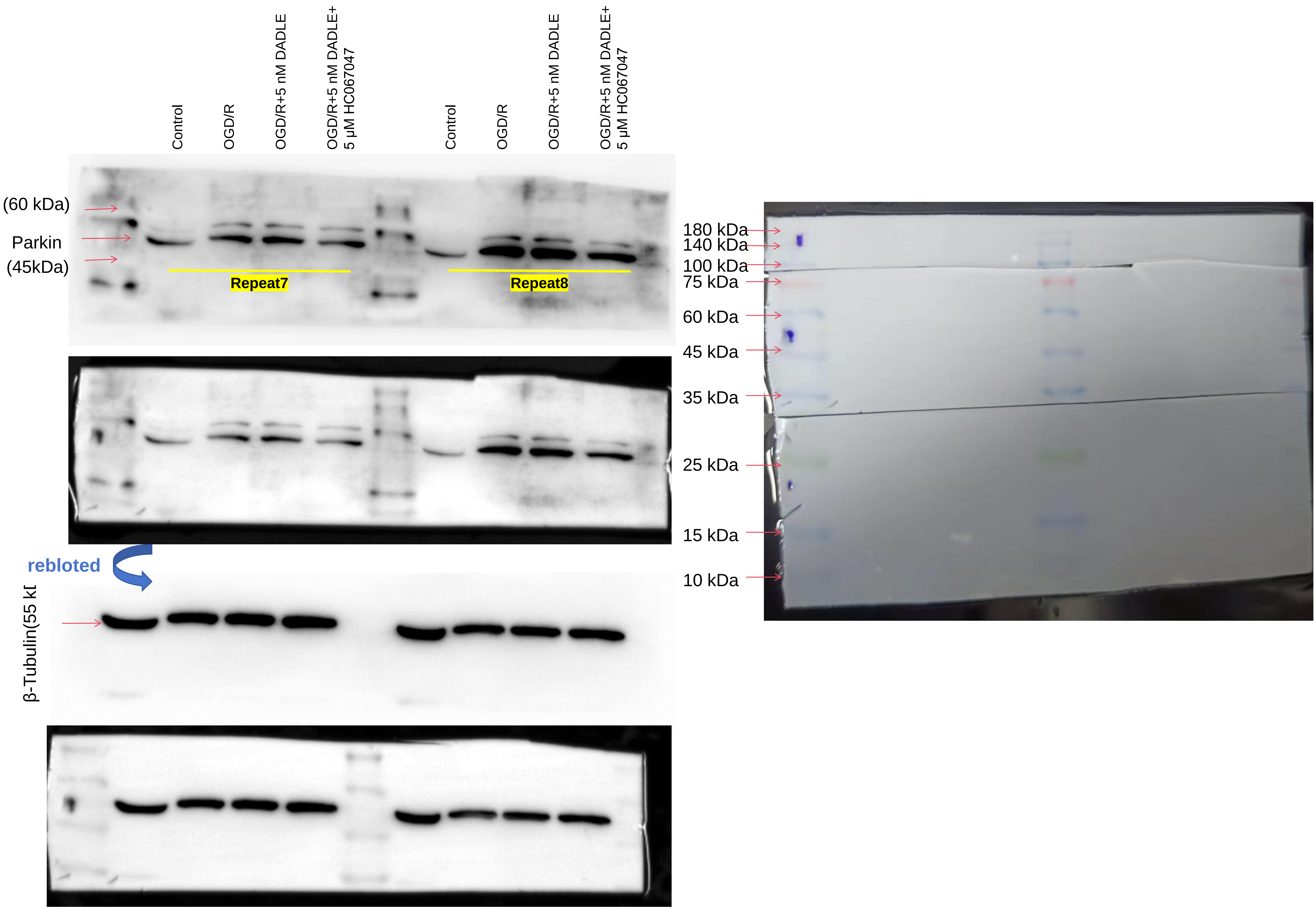
Control
OGD/R
OGD/R+5 nM DADLE
OGD/R+5 nM DADLE+
5 μM HC067047
Control
OGD/R
OGD/R+5 nM DADLE
OGD/R+5 nM DADLE+
5 μM HC067047
(60 kDa)
Parkin
(45kDa)
Repeat7
Repeat8
rebloted
β-Tubulin(55 k
180 kDa
140 kDa
100 kDa
75 kDa
60 kDa
45 kDa
35 kDa
25 kDa
15 kDa
10 kDa

Original wester blot for Figure 6A(TRPV4)

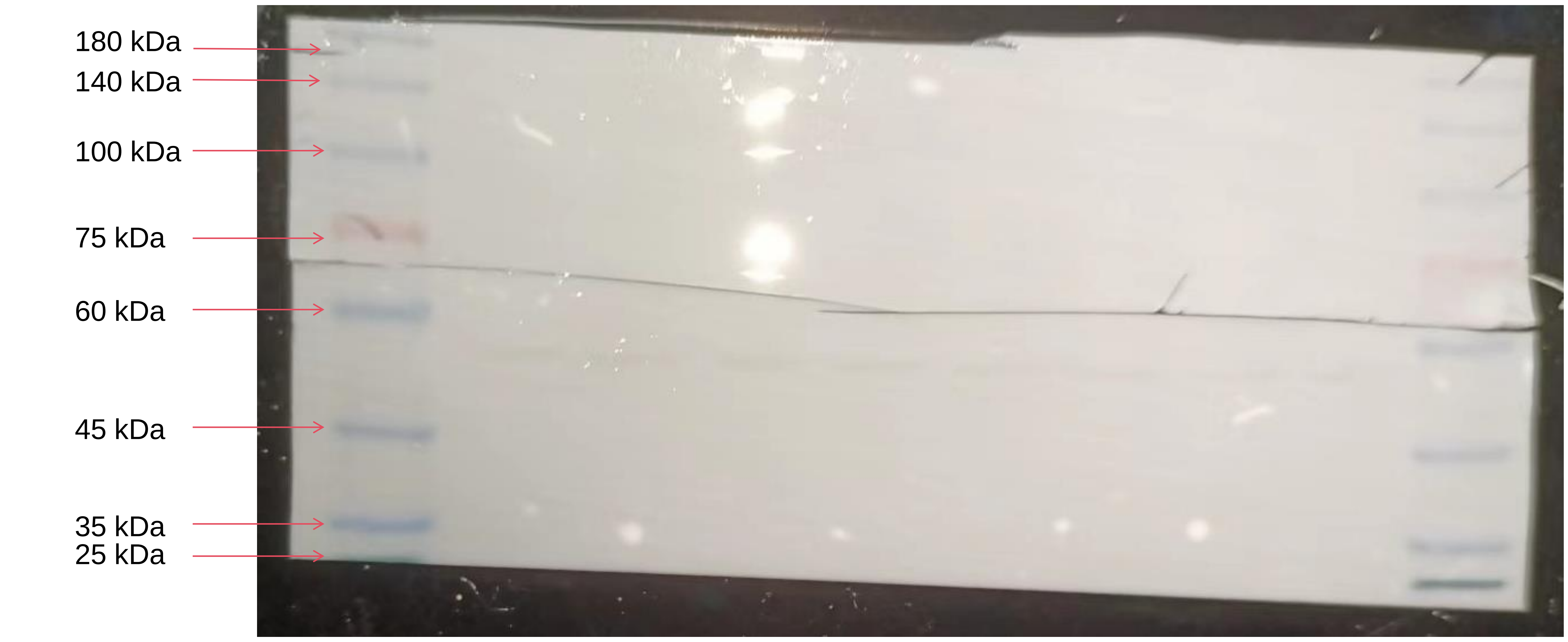

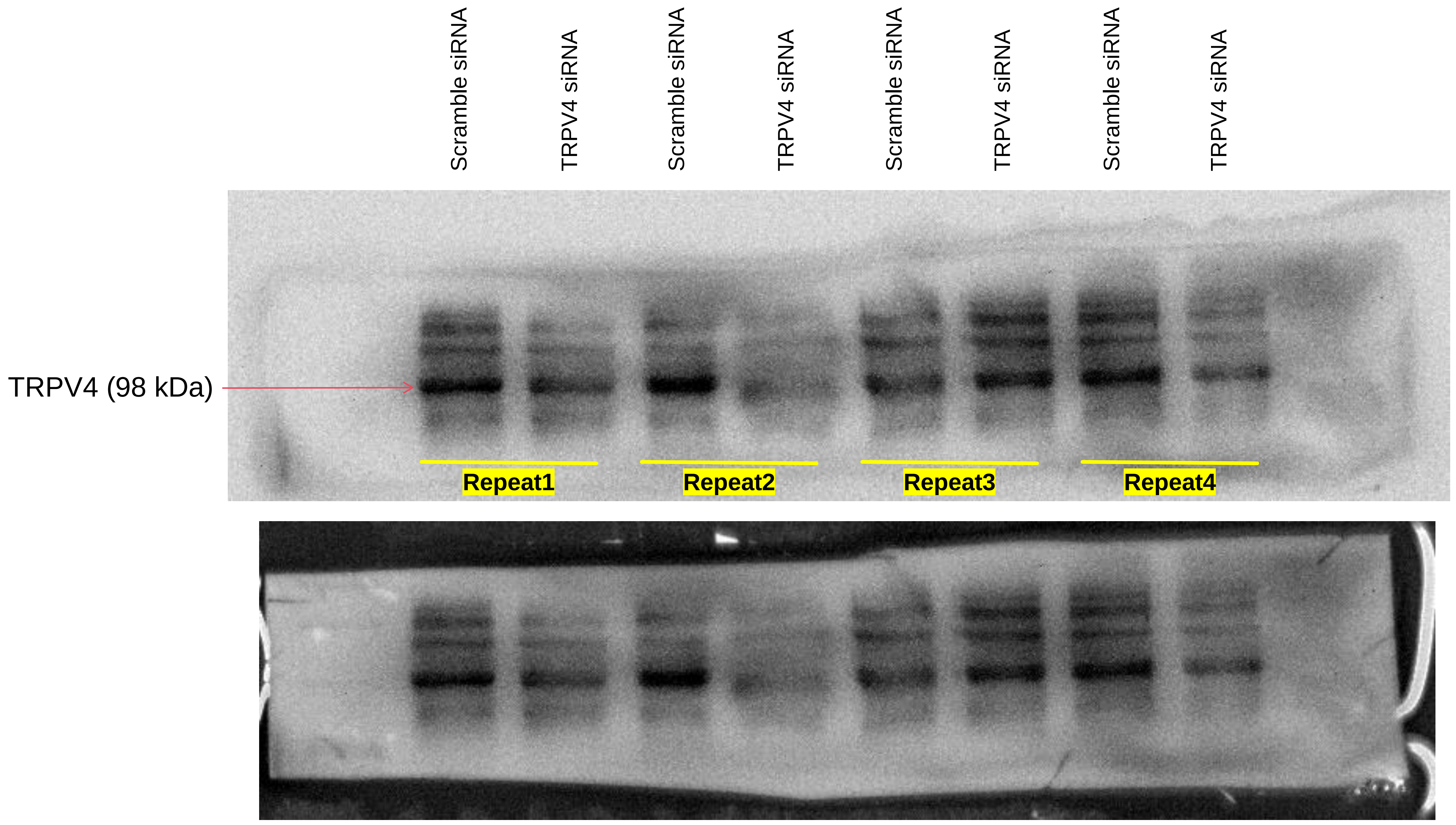

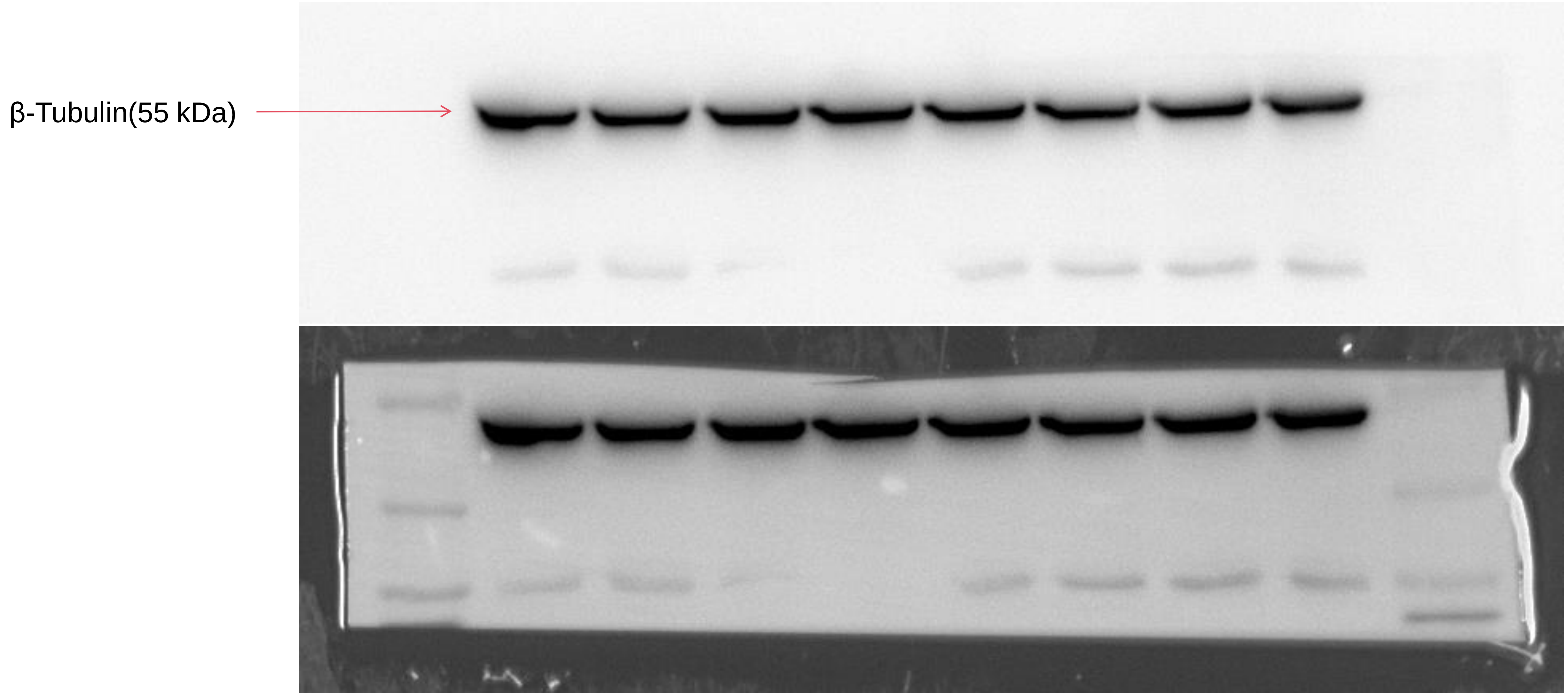

Original wester blot for Figure 6B(Beclin-1)

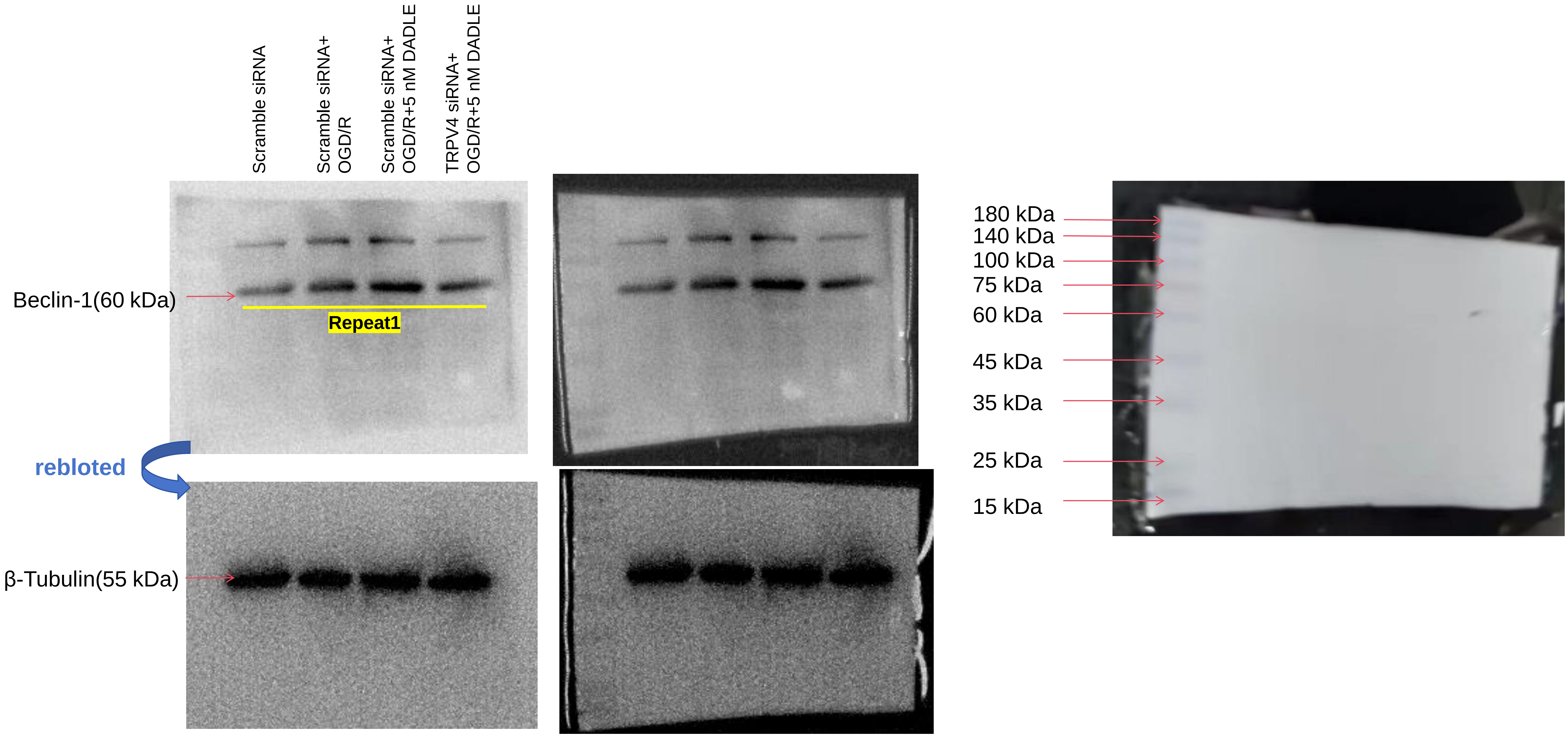

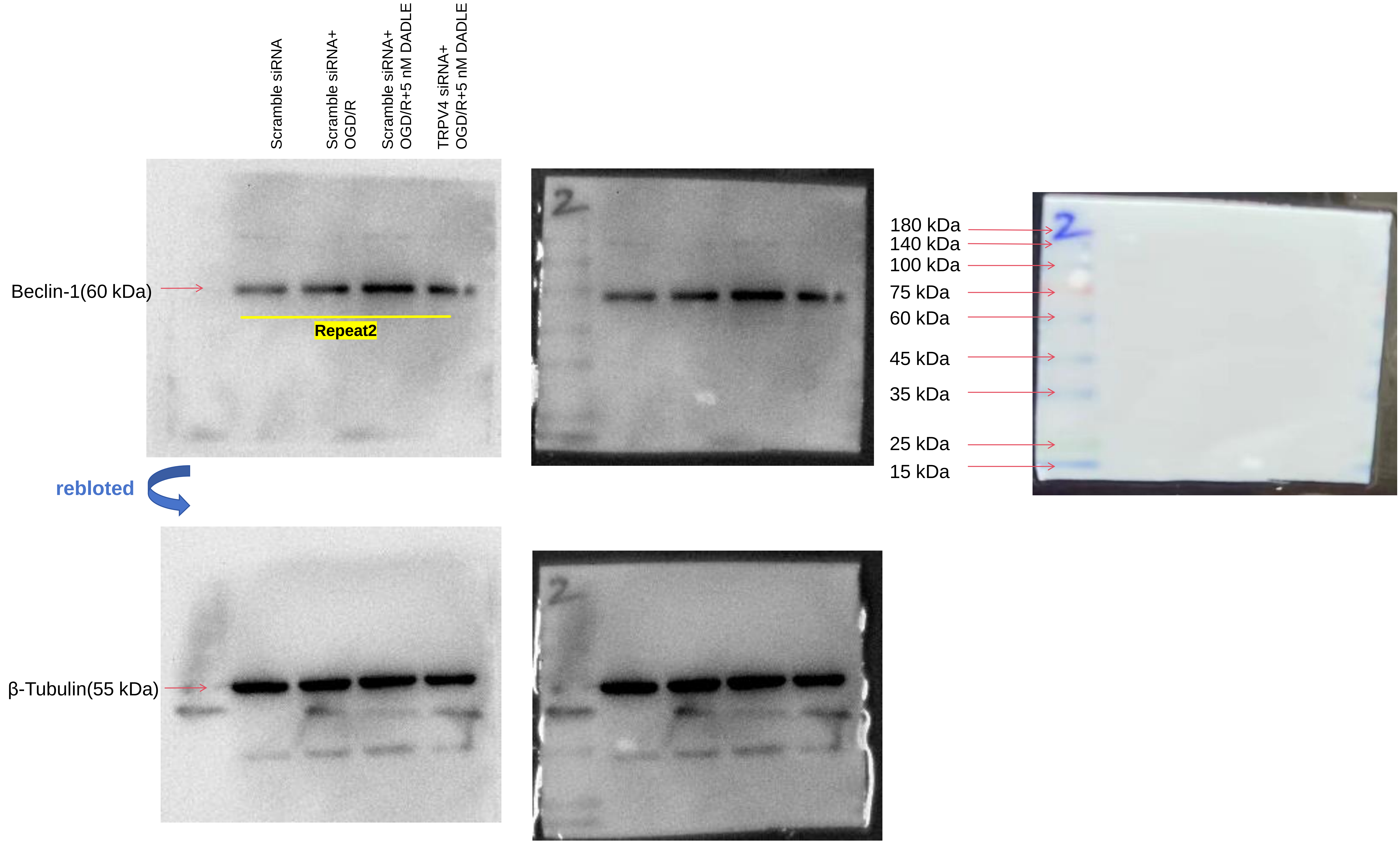

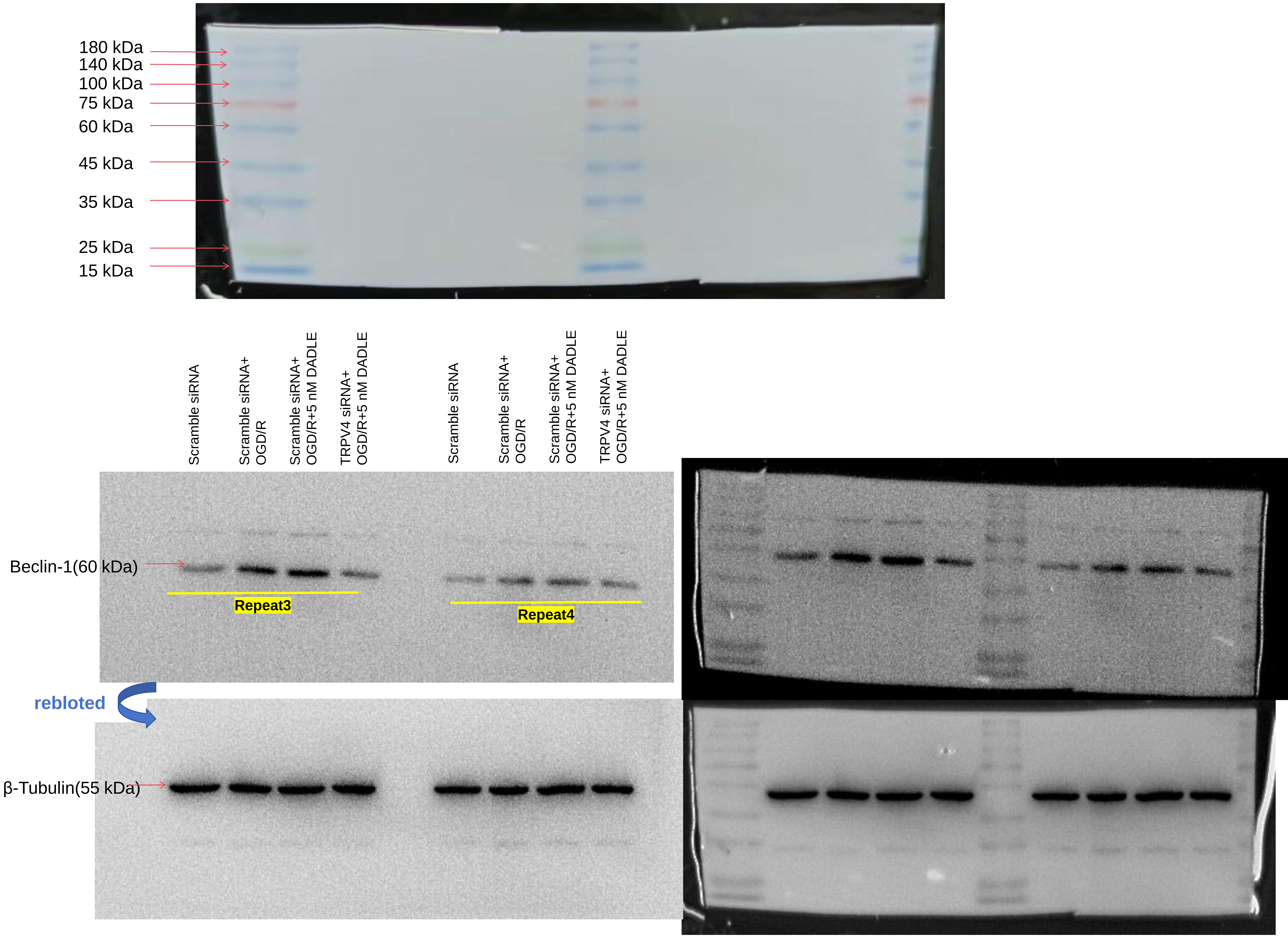

180 kDa
140 kDa
100 kDa
75 kDa
60 kDa
45 kDa
35 kDa
25 kDa
15 kDa
Scramble siRNA
Scramble siRNA+ OGD/R
Scramble siRNA+ OGD/R+5 nM DADLE
TRPV4 siRNA+ OGD/R+5 nM DADLE
Scramble siRNA
Scramble siRNA+ OGD/R
Scramble siRNA+ OGD/R+5 nM DADLE
TRPV4 siRNA+ OGD/R+5 nM DADLE
Beclin-1(60 kDa)
Repeat3
Repeat4
rebloted
β-Tubulin(55 kDa)

Original wester blot for Figure 6B(p62)

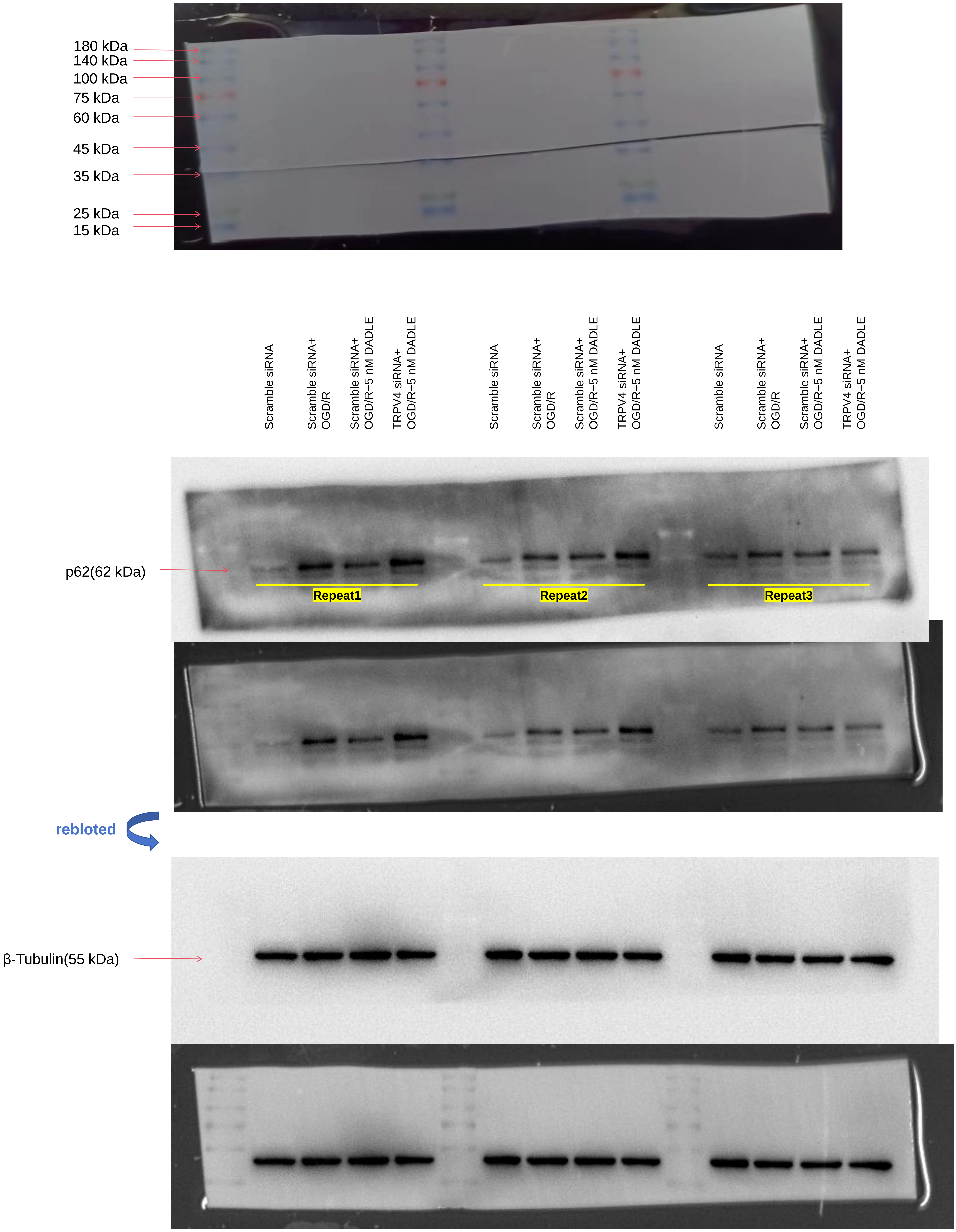

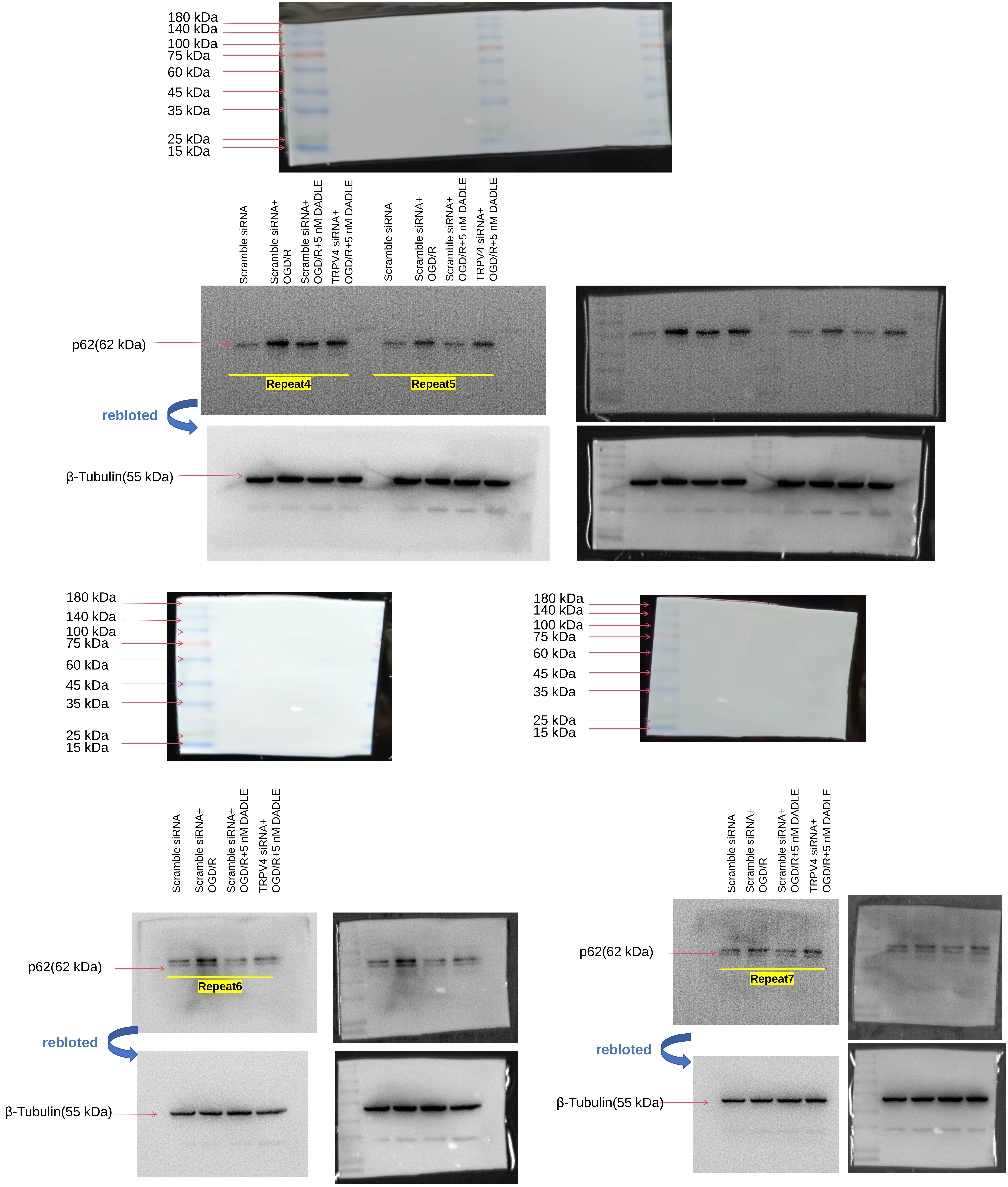

180 kDa
140 kDa
100 kDa
75 kDa
60 kDa
45 kDa
35 kDa
25 kDa
15 kDa
Scramble siRNA
Scramble siRNA+ OGD/R
Scramble siRNA+ OGD/R+5 nM DADLE
TRPV4 siRNA+ OGD/R+5 nM DADLE
Scramble siRNA
Scramble siRNA+ OGD/R
Scramble siRNA+ OGD/R+5 nM DADLE
TRPV4 siRNA+ OGD/R+5 nM DADLE
p62(62 kDa)
Repeat4
Repeat5
rebloted
β-Tubulin(55 kDa)
180 kDa
140 kDa
100 kDa
75 kDa
60 kDa
45 kDa
35 kDa
25 kDa
15 kDa
180 kDa
140 kDa
100 kDa
75 kDa
60 kDa
45 kDa
35 kDa
25 kDa
15 kDa
Scramble siRNA
Scramble siRNA+ OGD/R
Scramble siRNA+ OGD/R+5 nM DADLE
TRPV4 siRNA+ OGD/R+5 nM DADLE
p62(62 kDa)
Repeat6
rebloted
β-Tubulin(55 kDa)
Scramble siRNA
Scramble siRNA+ OGD/R
Scramble siRNA+ OGD/R+5 nM DADLE
TRPV4 siRNA+ OGD/R+5 nM DADLE
p62(62 kDa)
Repeat7
rebloted
β-Tubulin(55 kDa)

Original wester blot for Figure 6B(LC3Ⅱ、 Ⅰ)

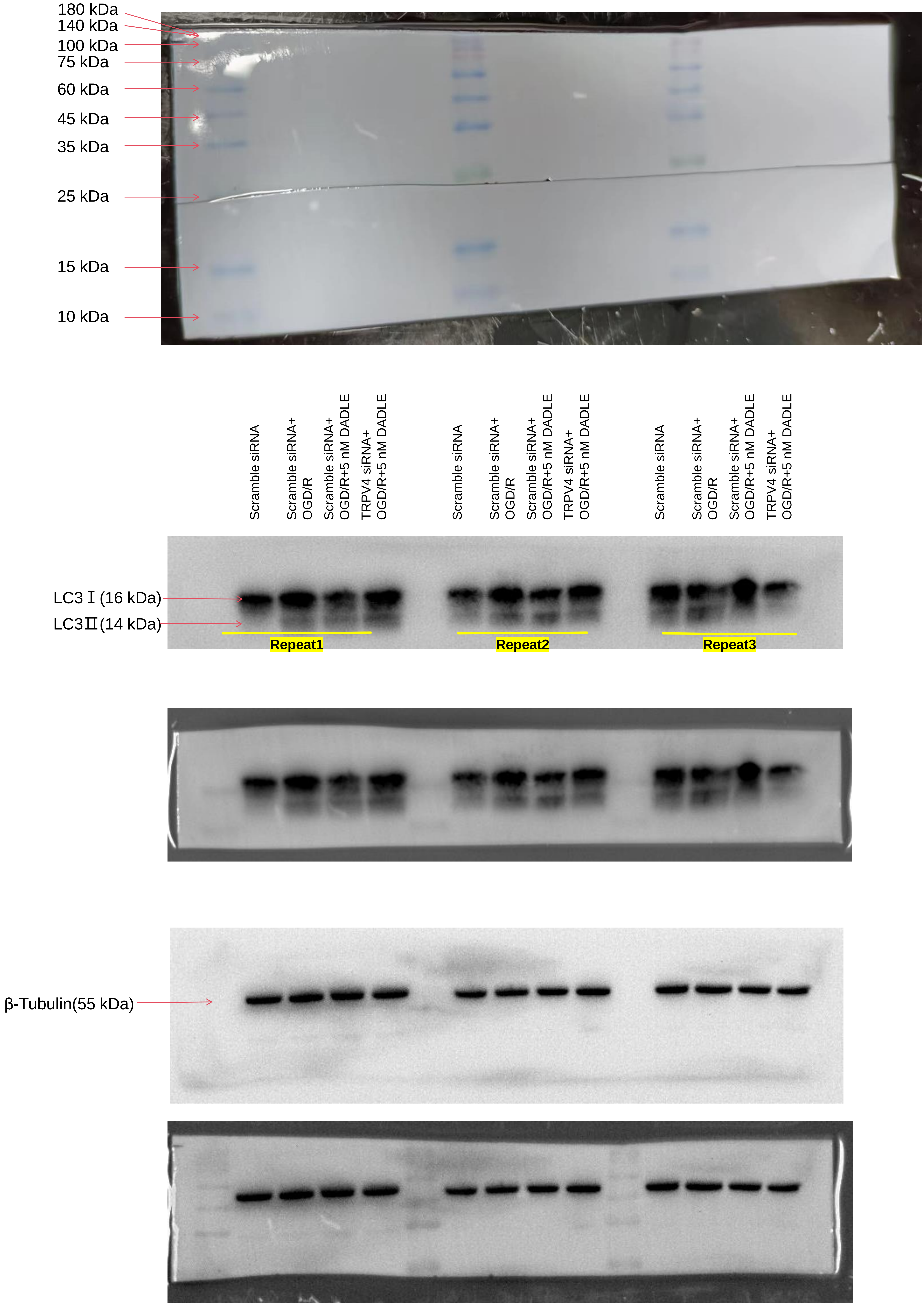

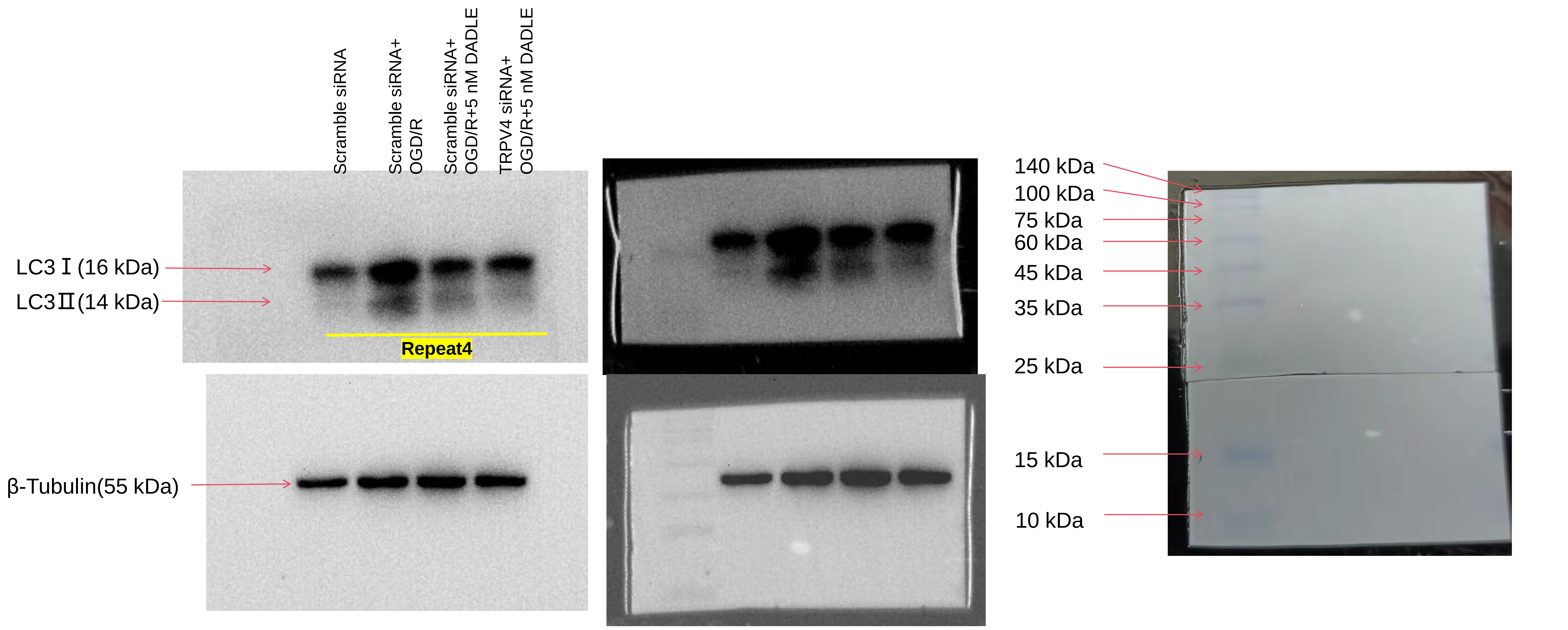
Scramble siRNA
Scramble siRNA+ OGD/R
Scramble siRNA+ OGD/R+5 nM DADLE
TRPV4 siRNA+ OGD/R+5 nM DADLE
LC3 I (16 kDa)
LC3 II (14 kDa)
Repeat4
β-Tubulin(55 kDa)
140 kDa
100 kDa
75 kDa
60 kDa
45 kDa
35 kDa
25 kDa
15 kDa
10 kDa

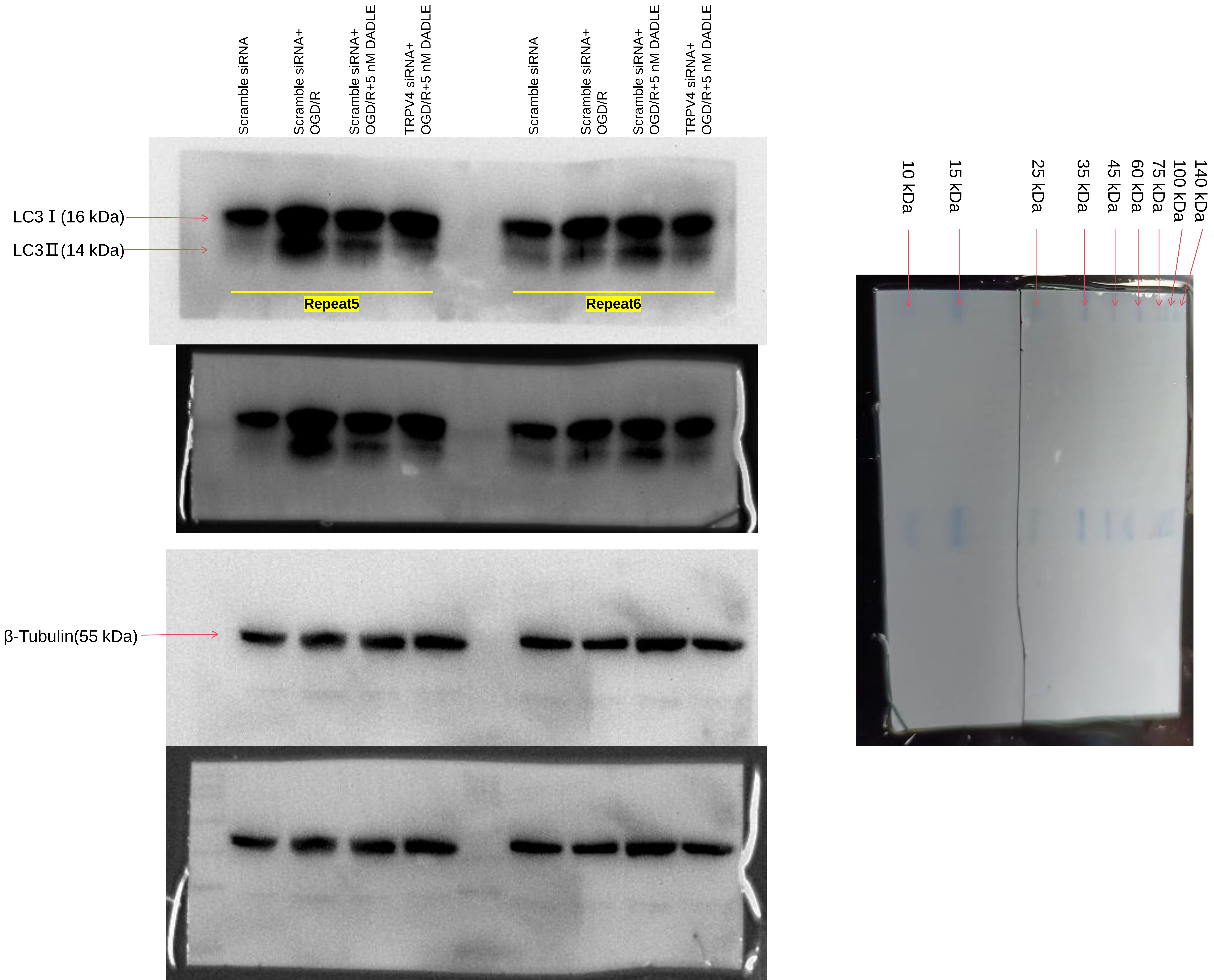
Scramble siRNA
Scramble siRNA+ OGD/R
Scramble siRNA+ OGD/R+5 nM DADLE
TRPV4 siRNA+ OGD/R+5 nM DADLE
Scramble siRNA
Scramble siRNA+ OGD/R
Scramble siRNA+ OGD/R+5 nM DADLE
TRPV4 siRNA+ OGD/R+5 nM DADLE
LC3 I (16 kDa)
LC3 II (14 kDa)
Repeat5
Repeat6
β-Tubulin(55 kDa)
10 kDa
15 kDa
25 kDa
35 kDa
45 kDa
60 kDa
75 kDa
100 kDa
140 kDa

Original wester blot for Figure 6B(PINK1)

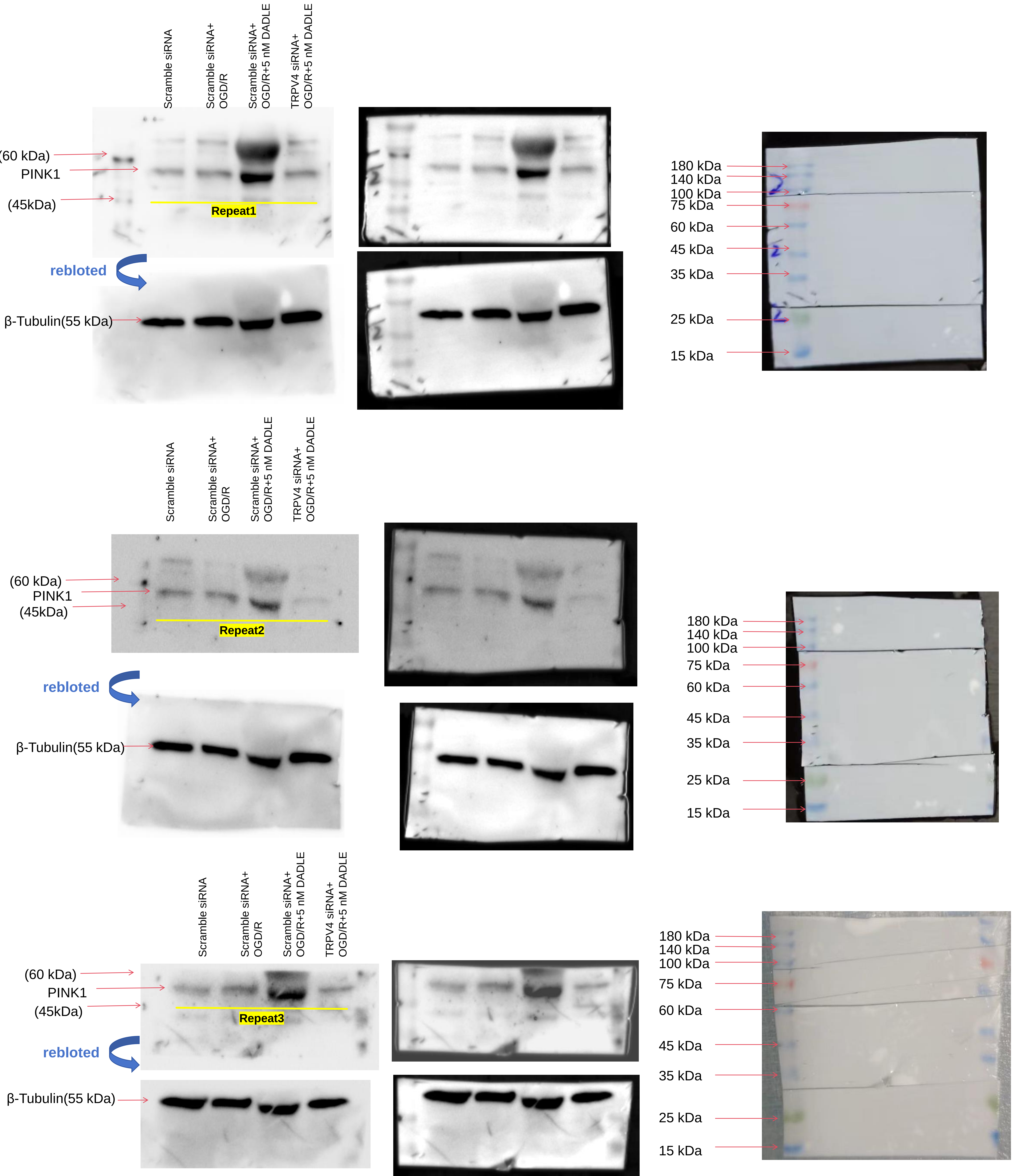

Original wester blot for Figure 6B(Parkin)

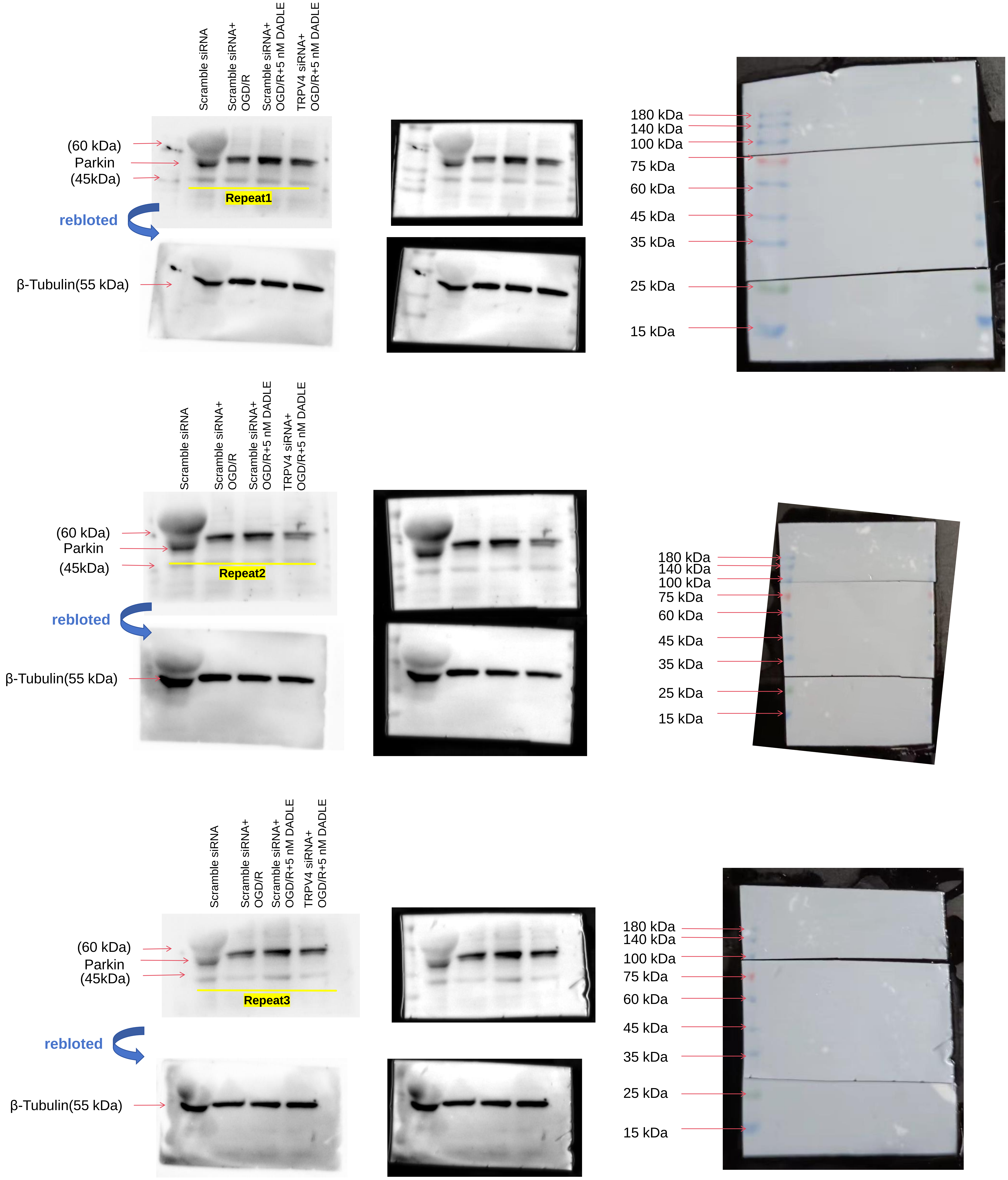

Supplement: online supplemental material 1 [file svn-10-1-s002.pdf]
